# Oral misoprostol (PGE1) *vs* vaginal dinoprostone (PGE2) for labor induction: individual participant data meta‐analysis of randomized controlled trials

**DOI:** 10.1002/uog.70100

**Published:** 2025-11-08

**Authors:** S. Y. Tan, N. Au, M. D. Peel, J. M. Dodd, A. Deussen, P. A. Le Roux, D. C. Young, F. Tessier, P. Dallenbäch, D. M. R. Croll, M. Patabendige, K. Palmer, W. Li, B. W. Mol

**Affiliations:** ^1^ Department of Obstetrics and Gynaecology Monash University Clayton VIC Australia; ^2^ Department of Obstetrics and Gynaecology Monash Health Clayton VIC Australia; ^3^ Department of Obstetrics and Gynaecology and Robinson Research Institute University of Adelaide, Women's and Children's Hospital North Adelaide SA Australia; ^4^ Women's and Babies Division, Women's and Children's Hospital North Adelaide SA Australia; ^5^ Department of Obstetrics and Gynaecology University of Cape Town Cape Town South Africa; ^6^ Department of Obstetrics and Gynaecology Dalhousie University, IWK Health Centre Halifax NS Canada; ^7^ Department of Obstetrics and Gynaecology University of British Columbia Vancouver BC Canada; ^8^ Department of Paediatrics, Gynaecology and Obstetrics Geneva University Hospital Geneva Switzerland; ^9^ Wilhelmina Children's Hospital Birth Centre University Medical Centre Utrecht Utrecht The Netherlands; ^10^ National Perinatal Epidemiology and Statistics Unit, Centre for Big Data Research in Health, Faculty of Medicine and Health University of New South Wales Sydney NSW Australia; ^11^ Aberdeen Centre for Women's Health Research University of Aberdeen Aberdeen UK; ^12^ Department of Obstetrics and Gynaecology Amsterdam University Medical Centre Amsterdam The Netherlands

**Keywords:** dinoprostone, individual participant data, induction of labor, misoprostol, PGE1, PGE2, safety, systematic review, vaginal delivery

## Abstract

**Objective:**

To compare the effectiveness and safety of oral misoprostol *vs* vaginal dinoprostone for the induction of labor (IOL) using an individual participant data (IPD) meta‐analysis.

**Methods:**

We used a Cochrane review and searched Ovid MEDLINE, Ovid Embase, Ovid Emcare, CINAHL Plus, Scopus and ClinicalTrials.gov to identify randomized controlled trials (RCTs) that compared oral misoprostol with vaginal dinoprostone for IOL in viable singleton pregnancies. We invited the authors of eligible trials to share their anonymized data. Primary outcomes were vaginal delivery, a composite measure of adverse maternal outcomes and a composite measure of adverse perinatal outcomes. IPD meta‐analysis was conducted using a two‐stage random‐effects model. An intention‐to‐treat approach was used for all analyses. Aggregate‐data meta‐analysis was undertaken with RCTs stratified by Trustworthiness in RAndomised Clinical Trials (TRACT) score.

**Results:**

Of 18 eligible RCTs, eight provided IPD, of which five (1892 participants) met the TRACT criteria. IPD meta‐analysis showed similar rates of vaginal delivery after IOL with oral misoprostol or vaginal dinoprostone (odds ratio (OR), 0.99 (95% CI, 0.80–1.22); *I* ^2^ = 0%). The rates of composite adverse perinatal outcome (adjusted odds ratio (aOR), 1.02 (95% CI, 0.61–1.72); *I* ^2^ = 0%) and composite adverse maternal outcome (aOR, 1.39 (95% CI, 0.72–2.69); *I* ^2^ = 0%) were also comparable between the groups. Of 10 RCTs that did not share IPD, seven met the TRACT criteria. Aggregate‐data meta‐analysis of the 12 RCTs (five with IPD and seven without IPD) meeting the trustworthiness criteria also showed comparable rates of vaginal delivery after oral misoprostol and after vaginal dinoprostone (OR, 1.08 (95% CI, 0.92–1.27)). In contrast, six studies not meeting the trustworthiness criteria (three with and three without IPD) reported a higher rate of vaginal delivery following oral misoprostol (OR, 1.34 (95% CI, 1.22–1.48)), resulting in an inflated overall estimate of the vaginal delivery rate after oral misoprostol based on all data (OR, 1.19 (95% CI, 1.05–1.36)).

**Conclusion:**

IOL with oral misoprostol or vaginal dinoprostone results in comparable rates of vaginal delivery and composite perinatal and maternal adverse outcomes. © 2025 The Author(s). *Ultrasound in Obstetrics & Gynecology* published by John Wiley & Sons Ltd on behalf of International Society of Ultrasound in Obstetrics and Gynecology.

## INTRODUCTION

Induction of labor (IOL), the process of starting labor artificially, is the most frequent obstetric intervention applied in Australia, occurring 100 000 times per year and affecting 35% of Australian pregnant women, with comparable rates elsewhere in the world^1^. In many obstetric situations, there is good evidence that IOL improves perinatal outcomes^2^, ^3^, ^4^, ^5^. Valid evidence on the effectiveness of IOL methods to achieve vaginal delivery and the safety of these methods for mother and baby is therefore essential.

The most commonly used pharmacological method for IOL is vaginal administration of prostaglandin E2 (PGE2, also known as dinoprostone)^6^. Prostaglandin E1 (PGE1, also known as misoprostol) is an alternative that can be administered orally or vaginally. The optimal route of administration of artificial prostaglandins is uncertain and has been widely debated^7^.

Several aggregate‐data meta‐analyses (AD‐MA) have shown that oral misoprostol and vaginal dinoprostone achieved similar rates of vaginal delivery and have comparable safety^7^, ^8^, ^9^. In a large network meta‐analysis, titrated low‐dose oral misoprostol was reported to achieve the lowest rate of Cesarean section^8^. However, owing to the rarity of adverse events and inconsistent reporting, AD‐MA is unable to adequately assess individual safety outcomes for this comparison. In addition, there is increasing concern about the trustworthiness of medical publications, including randomized controlled trials (RCTs)^10^.

Individual participant data meta‐analysis (IPD‐MA) has the potential to address these issues. Through IPD‐MA, data can be reanalyzed using the same statistical measures^11^, unreported outcomes can be evaluated and the integrity of the data can be verified^12^. Additionally, IPD‐MA enables thorough subgroup analysis and development of composite outcomes to measure safety^11^. Herein, we compare the effectiveness and safety of oral misoprostol *vs* vaginal dinoprostone for IOL using IPD‐MA methodology.

## METHODS

### Registration and ethical approval

This internationally collaborative IPD‐MA was registered prospectively in PROSPERO (registration number CRD42024479963) and was conducted in accordance with the Preferred Reporting Items for Systematic Reviews and Meta‐Analyses of Individual Participant Data (PRISMA‐IPD) guidelines^13^. Ethical approval was obtained from Monash University Human Research Ethics Committee (project ID 28883). Where the words ‘women’, ‘she’ and ‘her’ are used, it is to describe individuals whose sex was assigned at birth as female, whether they identify as female, male or non‐binary.

### Search strategy

We used the Cochrane review on oral misoprostol for IOL to identify eligible trials published prior to January 2014^8^. A subsequent search to identify trials published after this date was conducted in Ovid MEDLINE, Ovid Embase, Ovid Emcare, CINAHL Plus, Scopus and ClinicalTrials.gov and was completed on 8 July 2024.

Keywords used in the search strategy included ‘misoprostol’, ‘PGE1’, ‘dinoprostone’ and ‘PGE2’. These were paired with MeSH terms and Boolean operators to identify RCTs comparing oral misoprostol and vaginal dinoprostone for IOL (Table S1)

### Study selection

Eligible studies were RCTs that compared oral misoprostol and vaginal dinoprostone for IOL in women with a viable singleton gestation. No language restrictions were imposed, with assistance from online translation services and colleagues for non‐English‐language studies. We considered RCTs that included prelabor rupture of membranes (PROM) and were not selective regarding the dose of misoprostol or dinoprostone used. The online platform Covidence was used by two independent investigators (M.D.P., D.M.R.C.) to review titles and abstracts and then full‐text articles using the inclusion and exclusion criteria. In the event of disagreement, a third reviewer (B.W.M.) was consulted.

### Data access

We invited corresponding or primary authors to share raw data via e‐mail. Contact information was obtained from published articles, websites of associated institutions or networking websites, such as LinkedIn or ResearchGate. In the event of no response, we approached coauthors to participate via e‐mail. If there was still no response, institutions associated with the authors (including hospitals and universities), coauthors on more recent publications with the authors, colleagues from our network in the same country as the authors and the journal in which the eligible RCT was published were contacted to assist with finding the authors. A maximum of 10 rounds of e‐mail, WhatsApp message or WeChat message were sent per article.

Authors and corresponding institutions who agreed to share their data were asked to complete a data‐sharing agreement with our institution. After the agreement had been finalized and local ethics approval for data sharing had been obtained, data were anonymized by the original investigators and transferred through password‐protected worksheets via e‐mail. Data were checked for any missing or excluded data, errors, presence of randomization, internal consistency, reproducibility of the results from the published trial using the raw data and integrity^14^. Any discrepancies and inconsistencies were communicated with the original RCT investigators for a solution prior to inclusion in the current analysis. Any studies with concerns about data trustworthiness or unresolved issues were excluded from the final analysis.

### Outcomes

The primary outcomes were vaginal delivery, a composite of adverse maternal outcomes and a composite of adverse perinatal outcomes. The composite adverse maternal outcome included maternal death, admission to the intensive care unit, maternal infection (defined as a temperature of at least 38°C, antibiotic use at any time during labor or delivery, or a clinically diagnosed infection such as endometritis), severe postpartum hemorrhage ≥ 1000 mL and uterine rupture. The composite adverse perinatal outcomes included Apgar score < 7 at 5 min, stillbirth (defined as death of a fetus before birth), neonatal death (defined as death of a neonate within 28 days after birth), neonatal seizures, admission to the neonatal intensive care unit (NICU), admission to the NICU for > 48 h, severe neonatal respiratory compromise (including mechanical ventilation, infantile respiratory distress syndrome and pneumothorax) and meconium aspiration syndrome.

The effectiveness and safety profiles of misoprostol and dinoprostone for both mother and neonate were further assessed by secondary outcomes. These included mode of delivery (spontaneous vaginal delivery, assisted vaginal delivery, Cesarean section) and indication for Cesarean section. Outcomes to assess progression of labor included time from cervical ripening to vaginal delivery, oxytocin augmentation and use of analgesia during labor. Secondary maternal outcomes included the individual components of the composite adverse maternal outcome, plus uterine hyperstimulation (either tachysystole or hypertonus with a non‐reassuring fetal heart‐rate pattern on cardiotocography). Secondary perinatal outcomes included the individual components of the composite adverse perinatal outcome, plus meconium‐stained amniotic fluid.

### Assessment of risk of bias

Using the Cochrane risk of bias version 2 (RoB 2) tool^15^, two investigators (S.Y.T., M.P.) independently assessed the risk of bias of the included RCTs. Any discrepancies were discussed and resolved by consensus. The following domains were assessed: randomization process, deviations from intended interventions, missing outcome data, measurement of outcome and selection of the reported result, specifically related to the assignment to intervention.

### Trustworthiness assessment of studies that did not share IPD

For studies that did not share their raw data, the Trustworthiness in RAndomised Clinical Trials (TRACT) data integrity checklist tool was used to assess their trustworthiness (Table S2)^16^. The checklist aims to make an objective assessment of a study's trustworthiness by evaluating seven domains: governance, author group, plausibility of intervention, timeframe, dropout rates, baseline characteristics and outcomes. If needed, we contacted the original study authors for clarification. TRACT assessment was undertaken by two investigators independently (S.Y.T., M.P.). After applying the TRACT criteria to each study and taking into consideration the original study authors' response, identified concerns were discussed, after which a decision was made on trustworthiness. An arbitrary score of ≤ 8 was set as the threshold for meeting the trustworthiness criteria, and papers with a TRACT score of > 8 were therefore classified as not meeting the trustworthiness criteria.

### Data analysis and synthesis

An intention‐to‐treat analysis was performed for each outcome, using all available data. Oral misoprostol was considered as the experimental treatment, the comparator being vaginal dinoprostone.

Analysis was conducted by a two‐stage meta‐analysis. The first stage of the analysis compared oral misoprostol with vaginal dinoprostone for the predefined outcomes in each individual study. All models were adjusted for maternal age and parity. Adjusted odds ratios (aOR) with 95% CIs were calculated using logistic regression for binary outcomes. For continuous time‐to‐event outcomes, hazard ratios (HRs) and 95% CIs were calculated using a subdistribution hazard competing‐risks model, using Cesarean section and assisted vaginal delivery as competing risks. The second stage combined the results calculated from the first stage using a random‐effects model (restricted maximum likelihood estimator with Hartung–Knapp–Sidik–Jonkman method for 95% CI derivation). *I* ^2^ was used to quantify study heterogeneity.

A one‐stage method was used to analyze any outcome with no event in any group of any included study. Random‐effects logistic regression (using a stratified intercept and a random treatment effect, covariates with fixed effects and maximum likelihood estimator) adjusting for maternal age and parity was used in the one‐stage model.

Subgroup analyses of primary outcomes were performed using interaction terms between treatment and baseline covariates. Interactions were analyzed within each study and then pooled using random‐effects models to avoid ecologic bias. Subgroups reviewed included maternal age, parity, gestational age at IOL, maternal body mass index (BMI), initial Bishop score and PROM.

Meta‐analysis was conducted using Stata version 16.1 (StataCorp. LLC, College Station, TX, USA). The statistical package for social sciences (SPSS statistics for windows version 28.0; IBM Corp., Armonk, NY, USA) was used for summary statistics. Neither the public nor patients were involved in the planning of this IPD‐MA.

## RESULTS

### Study selection

**Figure 1:**
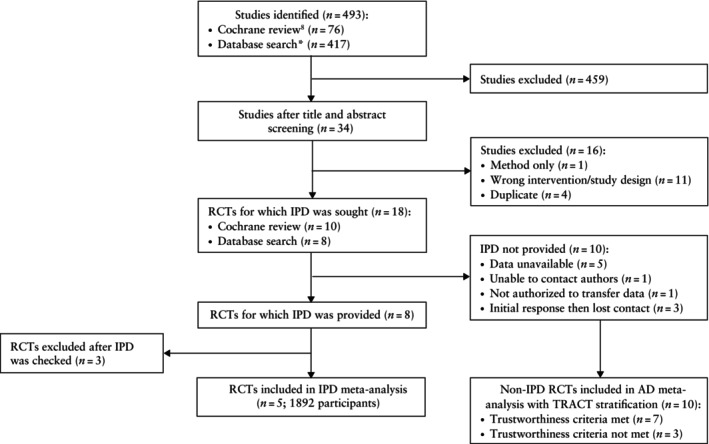
PRISMA individual participant data (IPD) flowchart summarizing inclusion of randomized controlled trials (RCTs) in meta‐analysis. *Search for RCTs published between 17 January 2014 and 8 July 2024. AD, aggregate data; TRACT, Trustworthiness in RAndomised Clinical Trials^16^.

We identified 10 RCTs from the Cochrane review on oral misoprostol for IOL (Figure 1)^8^. A systematic database search for more recently published papers retrieved an additional eight eligible RCTs. From these 18 eligible RCTs, the authors of eight studies were willing to share IPD. Authors of the other 10 studies did not share IPD for the following reasons: requested data were unavailable (*n* = 5)^17^, ^18^, ^19^, ^20^, ^21^, author group did not respond (*n* = 1)^22^, data were held in a clinical trial unit in the UK but it was not possible to get permission to transfer IPD (*n* = 1)^23^ and authors failed to respond to follow‐up despite an initial positive response (*n* = 3)^24^, ^25^, ^26^.

Of the eight RCTs that shared IPD, we decided not to use three owing to discrepancies between the data received and the published paper (Table S3). One study had identical strings in the spreadsheet resulting in duplicated data for more than 50% of patients^27^. In the second study, maternal age followed an unlikely distribution, and we could not replicate the published time‐to‐delivery results^28^. A third paper was excluded because the spreadsheet for the variable BMI contained a random number generator^29^. We reached out unsuccessfully to each author group for a response to the abovementioned concerns. Characteristics of all RCTs excluded from the IPD‐MA before or after data sharing are provided in Table S4.

### Study characteristics

Table 1 summarizes the methodology and inclusion/exclusion criteria of the five RCTs included in the final IPD‐MA. They were conducted in Australia^30^, Canada^31^, ^32^, South Africa^33^ and Switzerland^34^. The dose of oral misoprostol varied from 20 μg to 50 μg every 2–6 h, with different maximum amounts. The dose of vaginal dinoprostone was typically 1 mg or 2 mg every 6 h, up to a maximum of two doses. All RCTs included both nulliparous and parous women, and all but one excluded women with previous uterine surgery. Inclusion of women with PROM was allowed in three of the five trials^31^, ^32^, ^34^, and two trials included women from 34 weeks' gestation onwards^31^, ^33^.

**Table 1:** Characteristics of five randomized controlled trials included in individual participant data meta‐analysis

Study	Recruitment period	Country	Oral misoprostol protocol	Vaginal dinoprostone protocol	Inclusion criteria	Women (*n*)
Tessier (1997)^31^	05/1995 to 02/1996	Canada	50‐μg tablet every 6 h until established labor (max four doses)	2‐mg gel every 6 h until established labor (max four doses)	Any parity, ≥ 34 weeks' gestation, PROM allowed, BS NS, one previous CS allowed	267
Le Roux (2002)^33^	04/1999 to 11/2000	South Africa	50‐μg tablet every 6 h (max four doses)	1‐mg gel every 6 h (max two doses)	Any parity, ≥ 34 weeks' gestation, no PROM, BS ≤ 7, no previous CS	360
Dällenbach (2003)^34^	09/1999 to 04/2001	Switzerland	20 μg dissolved in water every 2 h, increased to 40 μg after 2 doses until established labor (max 10 40‐μg doses)	2‐mg gel every 6 h until BS ≥ 6 or established labor (max two doses)	Any parity, ≥ 37 weeks' gestation, PROM allowed, BS ≤ 6, no previous CS	202
Dodd (2006)^30^	04/2001 to 12/2004	Australia	20 μg dissolved in water, every 2 h (max six doses)	2‐mg gel (nulliparous) or 1‐mg gel (parous) every 6 h (max two doses)	Any parity, ≥ 36 + 6 weeks' gestation, no PROM, BS ≤ 6, no previous CS	724
Young (2020)^32^	04/1999 to 12/2000	Canada	50‐μg tablet every 4 h until progression of labor/three regular contractions in 10 min/non‐reassuring FHR or delivery (max NS)	1‐ or 2‐mg gel every 6 h until progression of labor/three regular contractions in 10 min/non‐reassuring FHR or delivery (max NS)	Any parity, ≥ 37 weeks' gestation, PROM allowed, BS NS, no previous CS	339

Only first author is given for each study. BS, Bishop score; CS, Cesarean section; FHR, fetal heart rate; max, maximum; NS, not stated; PROM, prelabor rupture of membranes.

### Risk of bias of included studies

**Figure 2:**
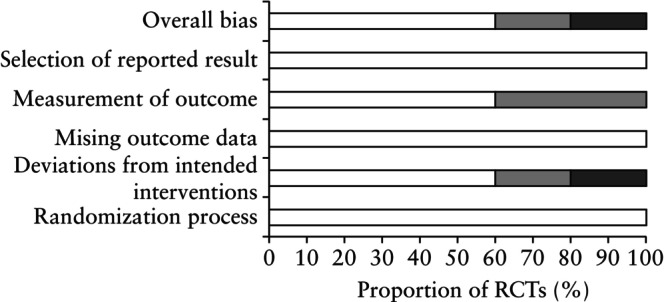
Risk‐of‐bias assessment of five randomized controlled trials (RCTs) included in intention‐to‐treat population. 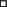, low risk; 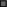, some concerns; 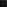, high risk.

On assessing the risk of bias, RCTs were mostly identified as low risk (Figures 2 and S1). Owing to the nature of the intervention, the lack of blinding of participants, caregivers and/or investigators caused multiple RCTs to be identified as having ‘some concerns’^34^ or as being at ‘high risk’ for bias^33^. Additionally, the unavailability of a prespecified analysis plan or lack of prospective trial registration also led to studies having ‘some concerns’ in the relevant domain^33^, ^34^. Four of the five RCTs were published prior to the introduction of mandatory trial registration^30^, ^31^, ^33^, ^34^.

### Characteristics of study participants

A total of 1892 women were analyzed, of whom 881 (46.6%) had been allocated to oral misoprostol and 1011 (53.4%) to vaginal dinoprostone. For one study, the data of 17 individual participants from one of the recruiting centers were not received, because separate ethical approval for data‐sharing could not be obtained^30^, hence the population size in this meta‐analysis was reduced accordingly. Baseline characteristics are shown for the overall population in Table 2 and for each individual study in Table S5. There were 1094 (57.9%) nulliparous women and 797 (42.1%) parous women; parity data were missing in one case.

**Table 2:** Baseline characteristics of participants in five randomized controlled trials included in individual participant data meta‐analysis

Characteristic	Overall (*n* = 1892)	Oral misoprostol (*n* = 881)	Vaginal dinoprostone (*n* = 1011)
Maternal age (years)*	28.7 ± 5.6	28.8 ± 5.6	28.5 ± 5.6
Maternal BMI (kg/m^2^)†	29.5 ± 6.8	29.4 ± 6.5	29.6 ± 7.1
Initial Bishop score‡	3.60 ± 1.77	3.52 ± 1.79	3.66 ± 1.76
Gestational age at IOL			
< 37 weeks (preterm)	78 (4.1)	36 (4.1)	42 (4.2)
37–40 weeks (term)	979 (51.7)	447 (50.7)	532 (52.6)
> 40 weeks (post‐term)	835 (44.1)	398 (45.2)	437 (43.2)
Nulliparous	1094/1891 (57.9)	515/880 (58.5)	579 (57.3)
Indication for IOL§			
> 1 indication	217 (11.5)	108 (12.3)	109 (10.8)
Antepartum hemorrhage	8 (0.4)	5 (0.6)	3 (0.3)
Decreased fetal movements	4 (0.2)	2 (0.2)	2 (0.2)
Diabetes mellitus	93 (4.9)	37 (4.2)	56 (5.5)
Elective/maternal request	11 (0.6)	6 (0.7)	5 (0.5)
Hypertensive disorder	362 (19.1)	158 (17.9)	204 (20.2)
Intrauterine growth restriction	62 (3.3)	28 (3.2)	34 (3.4)
Oligohydramnios	51 (2.7)	21 (2.4)	30 (3.0)
Post‐term gestation	737 (39.0)	350 (39.7)	387 (38.3)
Prelabor rupture of membranes	90 (4.8)	38 (4.3)	52 (5.1)
Other/unknown	257 (13.6)	128 (14.5)	129 (12.8)

Data are given as mean ± SD, *n* (%) or *n*/*N* (%). Smoking and ethnicity data were missing for all women.

* Data were missing for three women in oral misoprostol group and six women in vaginal dinoprostone group.

† Data were missing for 510 women in oral misoprostol group and 626 women in vaginal dinoprostone group.

‡ Data were missing for eight women in oral misoprostol group and nine women in vaginal dinoprostone group.

§ Counts are patients who experienced only that indication. BMI, body mass index; IOL, induction of labor.

### Synthesis of meta‐analysis

#### Primary outcomes

**Figure 3:**
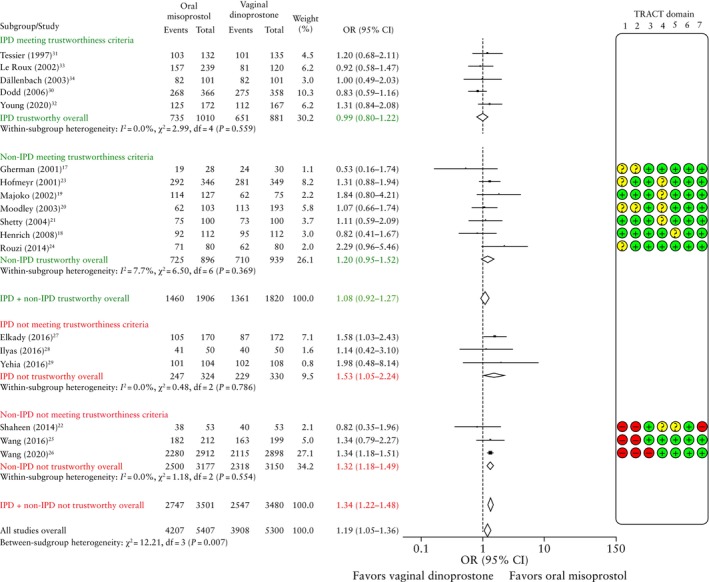
Forest plots comparing oral misoprostol *vs* vaginal dinoprostone for induction of labor according to rate of vaginal delivery, subdivided by availability of individual participant data (IPD) and Trustworthiness in RAndomised Controlled Trials (TRACT)^16^ assessment. Threshold for trustworthiness was set as TRACT score ≤ 8, where + indicates no concern, ? indicates some concern/no information and – indicates major concern. Only first author is shown for each study. Not adjusted for maternal age or parity. Weights are from random‐effects model. 95% CIs were derived by restricted maximum likelihood estimator with Hartung–Knapp–Sidik–Jonkman method. OR, odds ratio.

**Figure 4:**
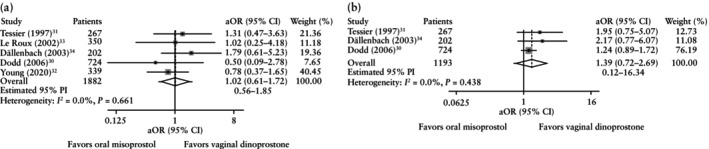
Forest plots from two‐stage meta‐analyses (adjusted for maternal age and parity) comparing oral misoprostol *vs* vaginal dinoprostone for induction of labor according to rate of composite adverse perinatal outcome (a) and composite adverse maternal outcome (b). Only first author is shown for each study. Weights (gray shading) are from random‐effects model. 95% CIs were derived by restricted maximum likelihood estimator with Hartung–Knapp–Sidik–Jonkman method. aOR, adjusted odds ratio; PI, prediction interval.

The primary outcome of vaginal delivery was reported by all five studies. The vaginal delivery rate was comparable between oral misoprostol and vaginal dinoprostone (OR, 0.99 (95% CI, 0.80–1.22); *I* ^2^ = 0.0%) (Figure 3). The rate of composite adverse perinatal outcome was also comparable between the two groups (aOR, 1.02 (95% CI, 0.61–1.72); *I* ^2^ = 0.0%) (Figure 4a). Only three of the five studies could be analyzed for the composite adverse maternal outcome owing to insufficient data from two studies^32^, ^33^. The rate of composite adverse maternal outcome was comparable between the two groups (aOR, 1.39 (95% CI, 0.72–2.69); *I* ^2^ = 0.0%) (Figure 4b).

#### Secondary outcomes

Secondary outcomes are displayed in Table 3 and Figures S2–S4. There was no statistical difference in the time‐to‐event analysis for the HR of spontaneous vaginal delivery between the two groups (five RCTs, 1864 women; subdistribution HR, 0.97 (95% CI, 0.93–1.01); *I* ^2^ = 92.7%). The rates of assisted vaginal delivery, Cesarean section for failure to progress or fetal distress, oxytocin and analgesia use, maternal infection, uterine hyperstimulation, severe postpartum hemorrhage, 5‐min Apgar score < 7, NICU admission and meconium‐stained amniotic fluid did not differ significantly between the groups. There was no case of stillbirth, neonatal or maternal death, maternal admission to the intensive care unit or uterine rupture in either group. Because of insufficient data on neonatal seizures, time spent in the NICU, severe neonatal respiratory compromise and meconium aspiration syndrome, these outcomes were not analyzed.

**Table 3:** Secondary outcomes comparing oral misoprostol and vaginal dinoprostone for induction of labor in five randomized controlled trials (RCTs) included in individual participant data meta‐analysis

Outcome	RCTs (*n* ^*ref*^)	Women (*n*)	Oral misoprostol*	Vaginal dinoprostone*	aOR (95% CI)†	*I* ^2^ (%) (*P*)‡	Analysis method
Delivery outcomes							
CS	5^30^, ^31^, ^32^, ^33^, ^34^	1882	273/1005 (27.16)	228/877 (26.00)	1.03 (0.80–1.32)	0.0 (0.625)	Two‐stage
CS for failure to progress	5^30^, ^31^, ^32^, ^33^, ^34^	1879	135/1003 (13.46)	115/876 (13.13)	1.10 (0.69–1.71)	21.7 (0.276)	Two‐stage
CS for fetal distress	5^30^, ^31^, ^32^, ^33^, ^34^	1879	101/1003 (10.07)	88/876 (10.05)	0.95 (0.69–1.31)	0.0 (0.713)	Two‐stage
Spontaneous VD	5^30^, ^31^, ^32^, ^33^, ^34^	1881	597/1004 (59.46)	517/877 (58.95)	1.01 (0.70–1.46)	34.4 (0.192)	Two‐stage
Assisted VD	4^30^, ^31^, ^32^, ^33^, ^34^	1158	71/639 (11.11)	68/519 (13.10)	1.01 (0.47–2.14)	27.3 (0.248)	Two‐stage
Labor progression outcomes							
Time to spontaneous VD	5^30^, ^31^, ^32^, ^33^, ^34^	1864	20.12 (12.04–28.21)	22.00 (12.15–31.85)	0.97 (0.93–1.01)§	92.7 (< 0.001)	Two‐stage
Oxytocin augmentation	5^30^, ^31^, ^32^, ^33^, ^34^	1882	383/1005 (38.11)	395/877 (45.04)	0.84 (0.38–1.83)	75.0 (0.003)	Two‐stage
Use of analgesia	4^31^, ^32^, ^33^, ^34^	1157	472/638 (73.98)	416/519 (80.15)	0.99 (0.65–1.51)	0.0 (0.600)	Two‐stage
Maternal adverse events							
Maternal infection	3^31^, ^32^, ^34^	808	13/405 (3.21)	11/403 (2.73)	1.05 (0.33‐3.34)	12.1 (0.320)	Two‐stage
Uterine hyperstimulation	4^30^, ^32^, ^33^, ^34^	1623	33/877 (3.76)	25/746 (3.35)	1.38 (0.79–2.43)	NA	One‐stage
Severe PPH	3^30^, ^31^, ^34^	1193	130/599 (21.70)	103/594 (17.34)	1.73 (0.39–7.56)	27.8 (0.251)	Two‐stage
Perinatal adverse events							
5‐min Apgar score < 7	5^30^, ^31^, ^32^, ^33^, ^34^	1892	15/1011 (1.48)	10/881 (1.14)	1.26 (0.37–4.25)	0.0 (0.416)	Two‐stage
NICU admission	4^30^, ^31^, ^33^, ^34^	1553	23/839 (2.74)	15/714 (2.10)	1.39 (0.60–3.21)	0.0 (0.630)	Two‐stage
Meconium‐stained AF	2^31^, ^34^	469	40/233 (17.17)	48/236 (20.34)	0.80 (0.08–7.66)	0.0 (0.458)	Two‐stage

* Data are given as *n*/*N* (%) or median (interquartile range).

† Adjusted for maternal age and parity.

‡ *P*‐value obtained from *t*‐test based on Hartung–Knapp–Sidik–Jonkman method.

§ Sub‐distribution hazard ratio. AF, amniotic fluid; aOR, adjusted odds ratio; CS, Cesarean section; NA, not applicable; NICU, neonatal intensive care unit; PPH, postpartum hemorrhage; VD, vaginal delivery.

#### Subgroup analysis

**Figure 5:**
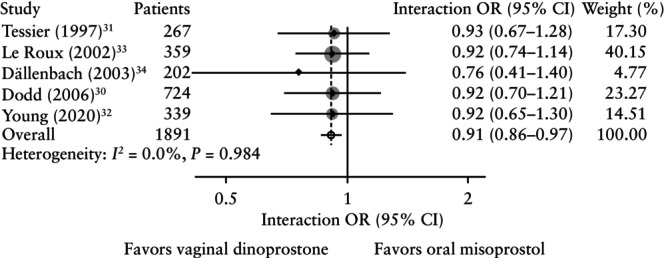
Forest plot from two‐stage subgroup analysis of interaction between gestational age and vaginal delivery when comparing oral misoprostol *vs* vaginal dinoprostone for induction of labor. Weights (gray shading) are from random‐effects model. 95% CIs were derived by restricted maximum likelihood estimator with Hartung–Knapp–Sidik–Jonkman method. OR, odds ratio.

For vaginal delivery, no interaction was identified for the subgroups maternal age, parity, maternal BMI, initial Bishop score or PROM (Table S6). However, a significant interaction between vaginal delivery and gestational age at IOL was identified (five RCTs, 1891 women; interaction OR, 0.91 (95% CI, 0.86–0.97); *I* ^2^ = 0.0%) (Figure 5). This indicates that for every 1‐week increase in gestational age, the odds of achieving vaginal delivery decrease by 9% when comparing oral misoprostol with vaginal dinoprostone for IOL. No significant interaction effects were observed for other primary outcomes.

#### Aggregate‐data meta‐analysis and IPD availability bias

The 10 RCTs that did not share IPD were assessed for trustworthiness, and were categorized as either meeting the trustworthiness criteria (TRACT score ≤ 8) or not meeting the trustworthiness criteria (TRACT score > 8) (Table S7)^16^. Studies not meeting the trustworthiness criteria lacked trial registration^22^, ^25^, ^26^, had a very large sample size compared with the number of authors^22^, ^26^, shared an author with a paper that has been retracted^25^, included author(s) who have published many RCTs^25^, ^26^, had an implausible timeframe^22^, demonstrated poor allocation concealment^26^, contained statistical mistakes^22^, ^25^, ^26^ and/or reported conflicting outcome data^22^. The largest study has since been retracted^26^.

Figure 3 shows AD‐MA for vaginal delivery stratified by the availability of IPD and trustworthiness. Among the non‐IPD RCTs that met our trustworthiness criteria (seven RCTs, 1835 women), there was no significant difference between oral misoprostol and vaginal dinoprostone for achieving vaginal delivery (OR, 1.20 (95% CI, 0.95–1.52); *I* ^2^ = 7.7%). The 12 RCTs meeting the trustworthiness criteria (five with IPD, seven without) reported an overall OR of 1.08 (95% CI, 0.92–1.27). Among the non‐IPD RCTs that did not meet our trustworthiness criteria (three RCTs, 6327 women), oral misoprostol showed a significantly higher rate of vaginal delivery compared with vaginal dinoprostone (OR, 1.32 (95% CI, 1.18–1.49); *I* ^2^ = 0.0%)^22^, ^25^, ^26^. The three RCTs that shared IPD but were considered non‐trustworthy also showed a significantly higher rate of vaginal delivery for oral misoprostol compared with vaginal dinoprostone (OR, 1.53 (95% CI, 1.05–2.24); *I* ^2^ = 0.0%)^27^, ^28^, ^29^.

The results of the overall meta‐analysis of all RCTs (IPD and no IPD, trustworthy and untrustworthy) revealed a higher rate of vaginal delivery with oral misoprostol compared to vaginal dinoprostone (18 RCTs, 10 707 women; OR, 1.19 (95% CI, 1.05–1.36)).

**Figure 6:**
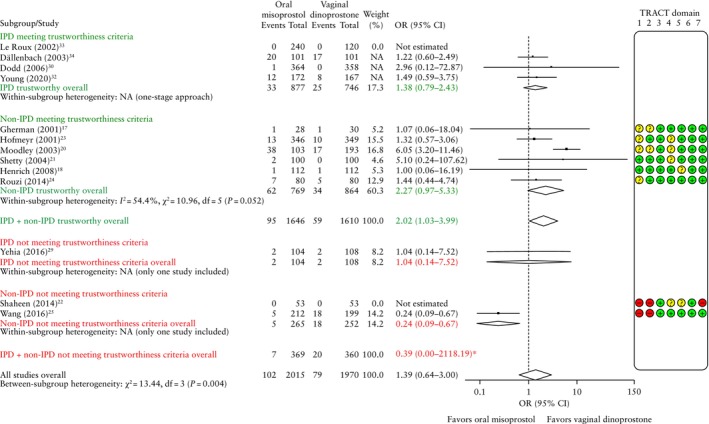
Forest plots comparing oral misoprostol *vs* vaginal dinoprostone for induction of labor according to rate of uterine hyperstimulation, subdivided by availability of individual participant data (IPD) and Trustworthiness in RAndomised Controlled Trials (TRACT)^16^ assessment. Threshold for trustworthiness was set as TRACT score ≤ 8, where + indicates no concern, ? indicates some concern/no information and – indicates major concern. Only first author is shown for each study. Not adjusted for maternal age or parity. Weights are from random‐effects model. 95% CIs were derived by restricted maximum likelihood estimator with Hartung–Knapp–Sidik–Jonkman method. *Odds ratio (OR) with 95% CI is not plotted owing to large difference in order of magnitude. NA, not applicable.

AD‐MA was also performed for individual maternal and perinatal adverse events. The overall meta‐analysis of IPD and non‐IPD RCTs that met the trustworthiness criteria indicated a significant increase in the rate of uterine hyperstimulation for oral misoprostol (OR, 2.02 (95% CI, 1.03–3.99)), whereas the overall meta‐analysis of IPD and non‐IPD RCTs that did not meet the trustworthiness criteria indicated no difference between vaginal dinoprostone and oral misoprostol for this outcome (OR, 0.39 (95% CI, 0.00–2118.19)) (Figure 6). AD‐MA for other secondary outcomes is shown in Figure S4.

### Publication bias

The funnel plot of all 18 eligible RCTs indicated a low risk of publication bias (Figure S5).

## DISCUSSION

### Main findings

Analysis of 1892 women undergoing IOL from five RCTs showed that oral misoprostol and vaginal dinoprostone result in comparable rates of vaginal delivery and composite perinatal and maternal adverse outcomes. The result for vaginal delivery was supported by AD‐MA of RCTs that met the TRACT criteria. RCTs not satisfying the trustworthiness criteria (three with and three without IPD) suggested incorrectly that oral misoprostol is associated with a higher rate of vaginal delivery.

### Strengths and limitations

The primary strength of this meta‐analysis is that it summarizes only IPD and papers reporting trustworthy data. The central coordinating team allowed continuous collaboration and communication with trial investigators to ensure the uniformity and integrity of the data. The predefined analysis and substantial sample size (1892 women) are additional strengths. It is unlikely that the findings were driven by a single RCT, because the largest study^30^ contributed only 38% of patients. Additionally, our IPD‐MA enabled outcome harmonization and treatment–covariate interaction analysis.

Our study also has limitations. Only five of the 18 potentially eligible studies were included in the final IPD analysis. Many excluded studies were published prior to 2010, making the lack of data‐sharing understandable. However, three of the eight raw datasets received were excluded owing to data integrity concerns^27^, ^28^, ^29^, and another three studies that did not provide IPD were also excluded for failing to meet the TRACT criteria^22^, ^25^, ^26^, one of which has since been retracted^26^. The lack of IPD from the remaining seven studies may introduce bias into the IPD‐MA. However, by evaluating all studies that did meet the trustworthiness criteria in an AD‐MA, we have shown that the findings were consistent. Second, using composite outcomes improves statistical power but leads to serious outcomes, such as maternal or perinatal death, being obscured by common outcomes. It is impractical to perform a sufficiently powered RCT to investigate individual adverse events; however, the use of composite outcomes is a solution for rare adverse events. We were unable to conduct an analysis of different dosing regimens because each study that shared IPD employed various dosing protocols for both drugs, limiting the potential for review on how this influenced the results. Lastly, the five studies included in the IPD analysis were performed in high‐resource settings, limiting the generalizability of our findings to low‐ and middle‐resource settings.

### Interpretation

This IPD‐MA showed that IOL with oral misoprostol and IOL with vaginal dinoprostone result in similar rates of vaginal delivery. This is contradictory to the AD‐MA of all 18 RCTs, which indicated that oral misoprostol was more likely to result in a vaginal delivery. As suggested by our IPD‐MA and AD‐MA, this difference may be driven by the inclusion of studies not meeting TRACT criteria that favored oral misoprostol. These findings highlight the need to interpret AD‐MAs with caution, as they are susceptible to error from problematic studies.

Composite adverse maternal and perinatal outcomes have not previously been commented on by AD‐MAs, as individual adverse outcomes have low event rates^8^. However, our IPD‐MA allowed for the combination of adverse effects, and indicated that both maternal and perinatal safety are comparable after oral misoprostol or vaginal dinoprostone. Stratified AD‐MA of studies meeting TRACT criteria also showed that oral misoprostol resulted in a higher rate of uterine hyperstimulation compared with vaginal dinoprostone. Maternal satisfaction was reported by only two of the RCTs that shared IPD and in different formats. Future studies should aim to review maternal satisfaction using a unified questionnaire, in addition to other maternal outcomes influenced by IOL. Considering that oral misoprostol and vaginal dinoprostone have comparable overall safety and vaginal delivery rates, oral misoprostol could be an appealing method for IOL in low‐to‐middle‐resource settings, because it is stable at room temperature, which is important when refrigeration of medications is not feasible. Additionally, oral misoprostol can be administered without a vaginal examination. This may improve overall maternal satisfaction with the IOL process, which is particularly important for women for whom vaginal examination might be traumatizing.

Vaginal dinoprostone, mechanical induction and oral misoprostol are currently recommended by the IOL guidelines of the Royal College of Obstetricians and Gynaecologists^35^, the American College of Obstetricians and Gynecologists^36^, the National Institute for Health and Care Excellence^6^ and the Royal Australian and New Zealand College of Obstetricians and Gynaecologists^37^. These recommendations are supported by this IPD‐MA. A previous IPD‐MA compared oral misoprostol to balloon catheters and showed a slightly increased risk of Cesarean section after IOL but better neonatal outcomes with the latter^38^. When compared with vaginal prostaglandins, balloon catheters result in a similar rate of Cesarean section and better neonatal outcomes^39^. There is a need for network IPD‐MA to identify the most safe and effective method of IOL.

Data reanalysis through data sharing has been increasingly recognized as a necessity in clinical research, with evidence indicating that between 25% and 40% of papers have concerns due to falsified data or are otherwise untrustworthy^10^. This percentage was confirmed in our meta‐analysis, in which we excluded three RCTs (654 participants) after reviewing the raw data, and another three RCTs (6327 participants) for failing to meet the TRACT criteria (33% of studies, 66% of participants). One of the excluded RCTs has since been retracted^26^; this study had a TRACT score of 10, indicating that a cut‐off for trustworthiness of ≤ 8 was justified. From 1 July 2018, the International Committee of Medical Journal Editors has enforced the inclusion of data‐sharing statements, believing it to be an ethical obligation, as participants put themselves at risk by partaking in trials^40^. Reasons behind not sharing data include the potential challenge it may pose to the study's trustworthiness or a lack of data to support the study. In addition to ensuring data validity, data sharing also allows further analysis of larger sample sizes, which strengthens and increases the value of the original research and the risks that participants undertook during those trials^41^. While our effort to highlight the discrepancies in three datasets may discourage authors from sharing their data in future analyses, we believe that it is important to be transparent about our findings. Data not meeting trustworthiness criteria can compromise the results of systematic reviews, as demonstrated in our analysis. Moreover, we gave authors the opportunity to respond to our findings, but they declined to do so^27^, ^28^, ^29^.

### Conclusions

This IPD‐MA indicates that oral misoprostol and vaginal dinoprostone are comparable for IOL with regard to rate of vaginal delivery, perinatal safety and maternal safety. This evidence should help to inform IOL guidelines and assist in decision‐making for both clinicians and patients.

## Supporting information


**Table S1** Search strategy.
**Table S2** Trustworthiness in RAndomised Clinical Trials (TRACT) scoring system.
**Table S3** Details of randomized controlled trials excluded from individual participant data (IPD) meta‐analysis due to discrepancy between IPD received and published study.
**Table S4** Characteristics of randomized controlled trials for which enquiry for individual participant data (IPD) was sent but did not contribute to IPD meta‐analysis.
**Table S5** Baseline characteristics of participants in randomized controlled trials included in individual participant data meta‐analysis, stratified by individual study.
**Table S6** Subgroup analysis for vaginal delivery (vaginal dinoprostone *vs* oral misoprostol) and composite adverse maternal outcome and composite adverse perinatal outcome (oral misoprostol *vs* vaginal dinoprostone).
**Table S7** Trustworthiness in RAndomised Clinical Trials (TRACT) scores for randomized control studies that did not share individual participant data.
**Figure S1** Risk‐of‐bias assessment for randomized controlled trials that provided individual participant data and were included in meta‐analysis.
**Figure S2** Forest plots from two‐stage meta‐analyses (adjusted for maternal age and parity) comparing oral misoprostol *vs* vaginal dinoprostone for induction of labor according to rate of secondary outcomes.
**Figure S3** Stacked cumulative proportion plot of mode of delivery over time from labor induction to delivery. *x*‐axis represents time (in h) on a logarithmic scale. *y*‐axis represents cumulative percentage of deliveries.
**Figure S4** Forest plots comparing oral misoprostol *vs* vaginal dinoprostone for induction of labor according to rate of secondary outcomes, subdivided by individual participant data (IPD) and Trustworthiness in RAndomised Controlled Trials (TRACT) assessment. Threshold for trustworthiness was set as TRACT score ≤ 8, where + indicates no concern, ? indicates some concern/no information and – indicates major concern. Only first author is shown for each study. Not adjusted for maternal age or parity. Weights are from random‐effects model.
**Figure S5** Funnel plot for primary outcome in all 18 randomized control trials for which individual participant data was requested.

## Data Availability

Data available on reasonable request, and after approval of all the individual authors who shared original data.
