# Supplementary material for: Oral misoprostol (PGE1) vs vaginal dinoprostone (PGE2) for labor induction: individual participant data meta‐analysis of randomized controlled trials
Source: Ultrasound Obstet Gynecol. 2025 Nov 8;67(5):579–89. doi: 10.1002/uog.70100 (PMC13136056; doi:10.1002/uog.70100)
Supplement: Supplementary file 1 — Table S1 Search strategy. Table S2 Trustworthiness in RAndomised Clinical Trials (TRACT) scoring system. Table S3 Details of randomized controlled trials excluded from individual participant data (IPD) meta‐analysis due to discrepancy between IPD received and published study. Table S4 Characteristics of randomized controlled trials for which enquiry for individual participant data (IPD) was sent but did not contribute to IPD meta‐analysis. Table S5 Baseline characteristics of participants in randomized controlled trials included in individual participant data meta‐analysis, stratified by individual study. Table S6 Subgroup analysis for vaginal delivery (vaginal dinoprostone vs oral misoprostol) and composite adverse maternal outcome and composite adverse perinatal outcome (oral misoprostol vs vaginal dinoprostone). Table S7 Trustworthiness in RAndomised Clinical Trials (TRACT) scores for randomized control studies that did not share individual participant data. Figure S1 Risk‐of‐bias assessment for randomized controlled trials that provided individual participant data and were included in meta‐analysis. Figure S2 Forest plots from two‐stage meta‐analyses (adjusted for maternal age and parity) comparing oral misoprostol vs vaginal dinoprostone for induction of labor according to rate of secondary outcomes. Figure S3 Stacked cumulative proportion plot of mode of delivery over time from labor induction to delivery. x‐axis represents time (in h) on a logarithmic scale. y‐axis represents cumulative percentage of deliveries. Figure S4 Forest plots comparing oral misoprostol vs vaginal dinoprostone for induction of labor according to rate of secondary outcomes, subdivided by individual participant data (IPD) and Trustworthiness in RAndomised Controlled Trials (TRACT) assessment. Threshold for trustworthiness was set as TRACT score ≤ 8, where + indicates no concern, ? indicates some concern/no information and – indicates major concern. Only first author is shown for each st [file UOG-67-579-s001.docx]

## Supplementary Figures

Figure S1: Risk-of-bias assessment for randomized controlled trials that provided individual participant data and were included in meta-analysis

| Study | D1 | D2 | D3 | D4 | D5 | | Overall |
| --- | --- | --- | --- | --- | --- | --- | --- |
| Dallenbach 2003 |  |  |  |  |  | |  |
| Dodd 2006 |  |  |  |  |  | |  |
| Le Roux 2002 |  |  |  |  |  | |  |
| Tessier 1997 |  |  |  |  |  | |  |
| Young 2020 |  |  |  |  |  | |  |
|  | D1: Randomisation process D2: Deviations from the intended intervention D3: Missing outcome data D4: Measurement of the outcome D5: Selection of the reported result | | | | | \| Judgement  High \| \| --- \|   Some concerns  Low | |
|  |  |  |  |  |  |  |  |
|  |  |  |  |  |  |  |  |
|  |  |  |  |  |  |  |  |
|  |  |  |  |  |  |  |  |

Figure S2: Forest plots from two-stage meta-analyses (adjusted for maternal age and parity) comparing oral misoprostol *vs* vaginal dinoprostone for induction of labor according to rate of secondary outcomes.

Figure S2A: Cesarean delivery for failure to progress


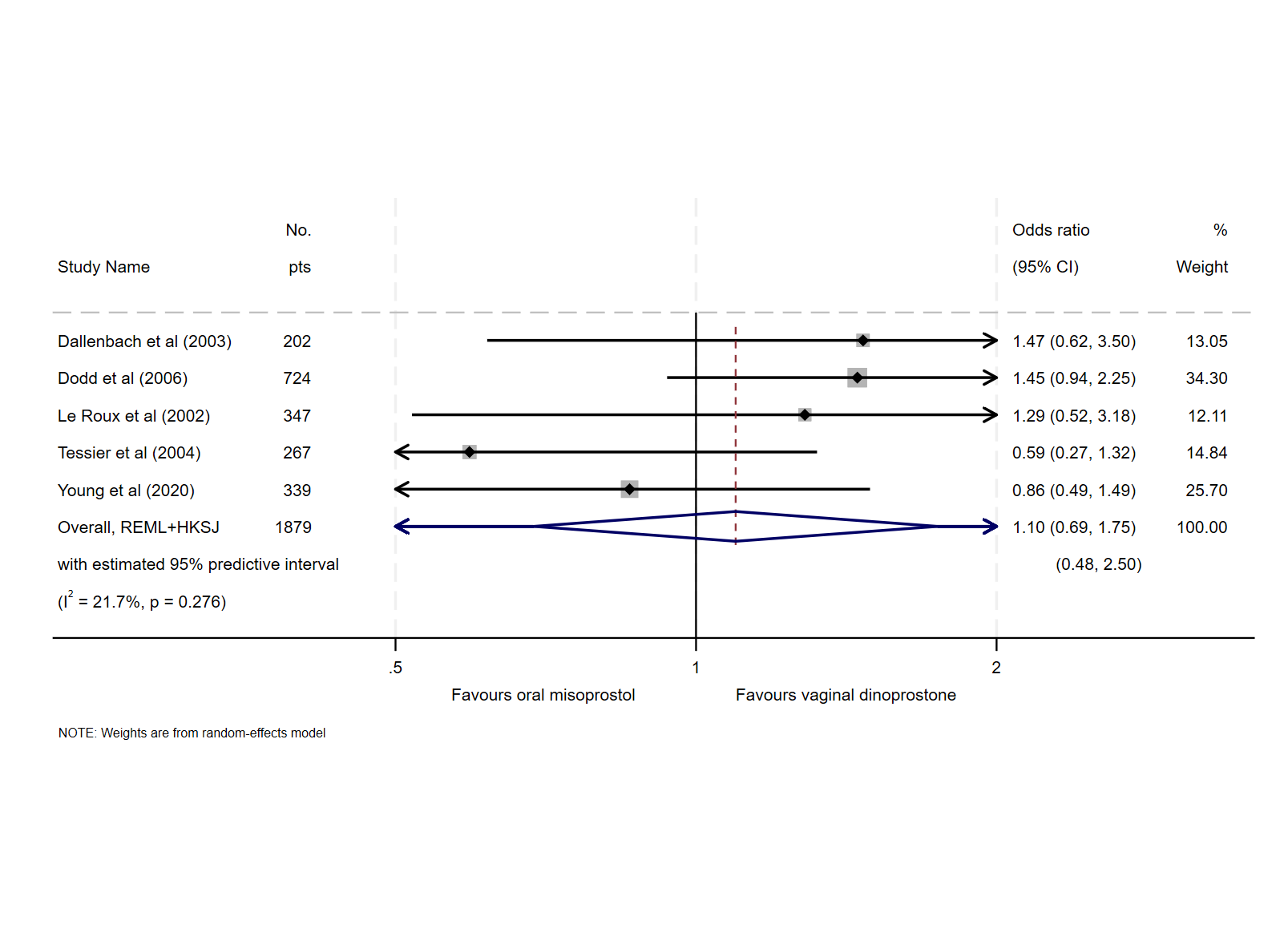


Figure S2B: Cesarean delivery for fetal distress


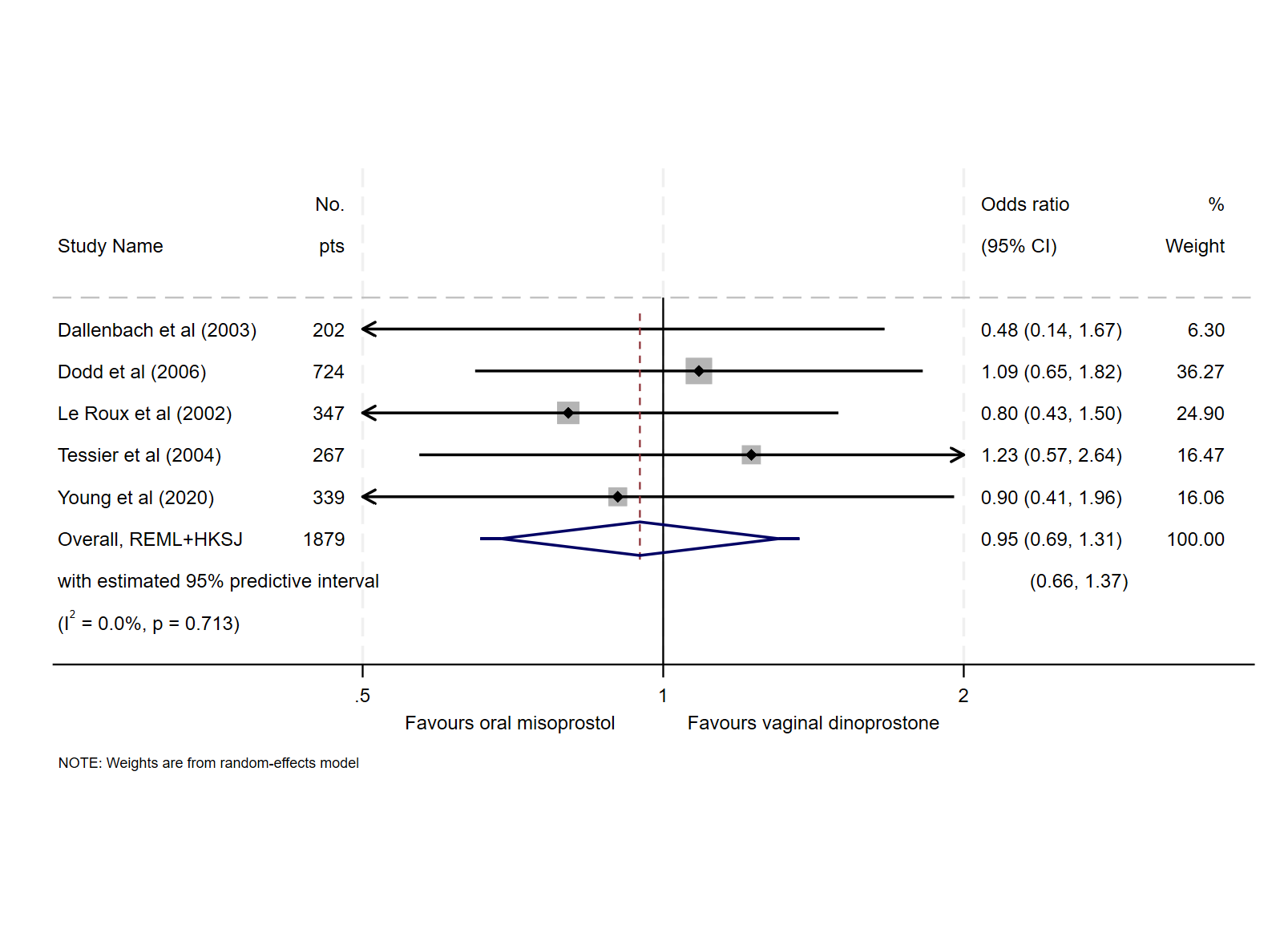


Figure S2C: Unassisted vaginal delivery


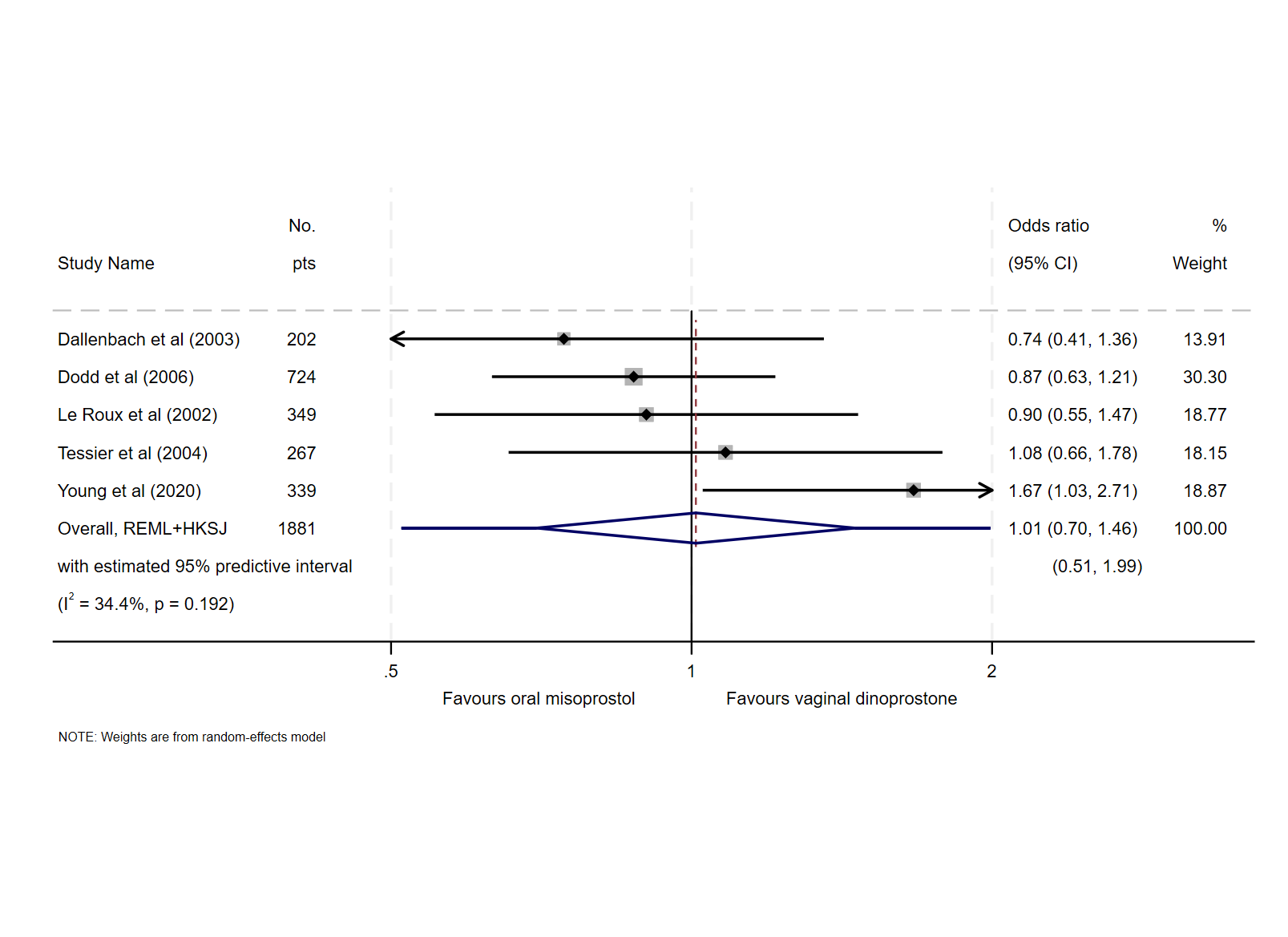


Figure S2D: Instrumental vaginal delivery


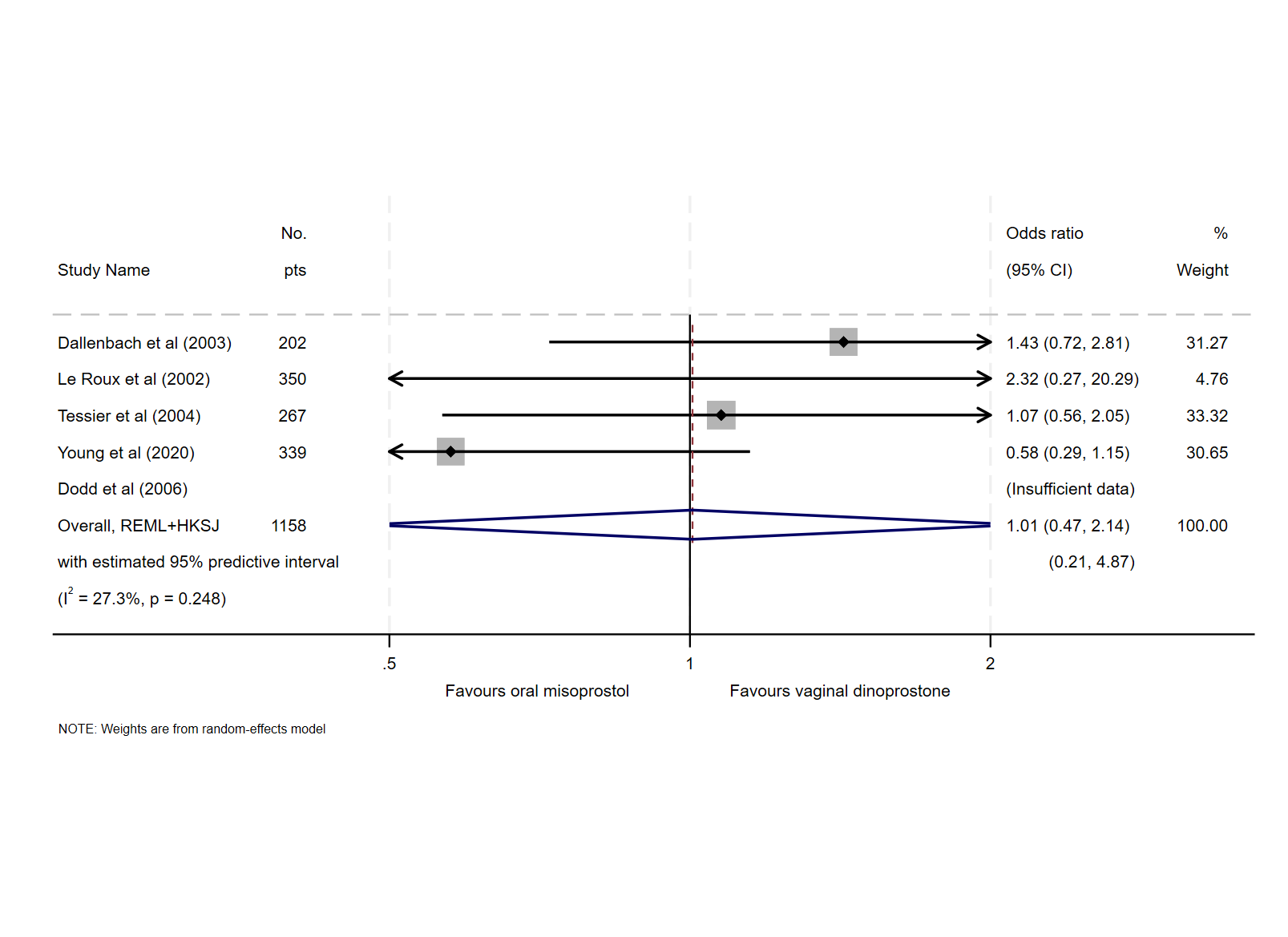


Figure S2E: Oxytocin Augmentation


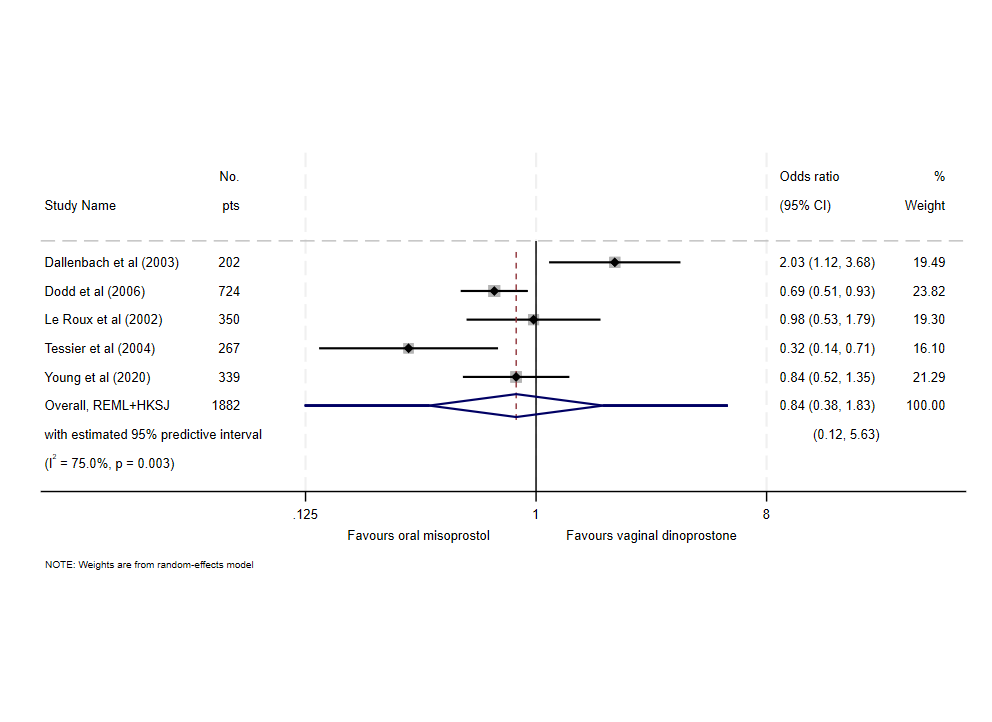


Figure S2F: Use of Analgesia


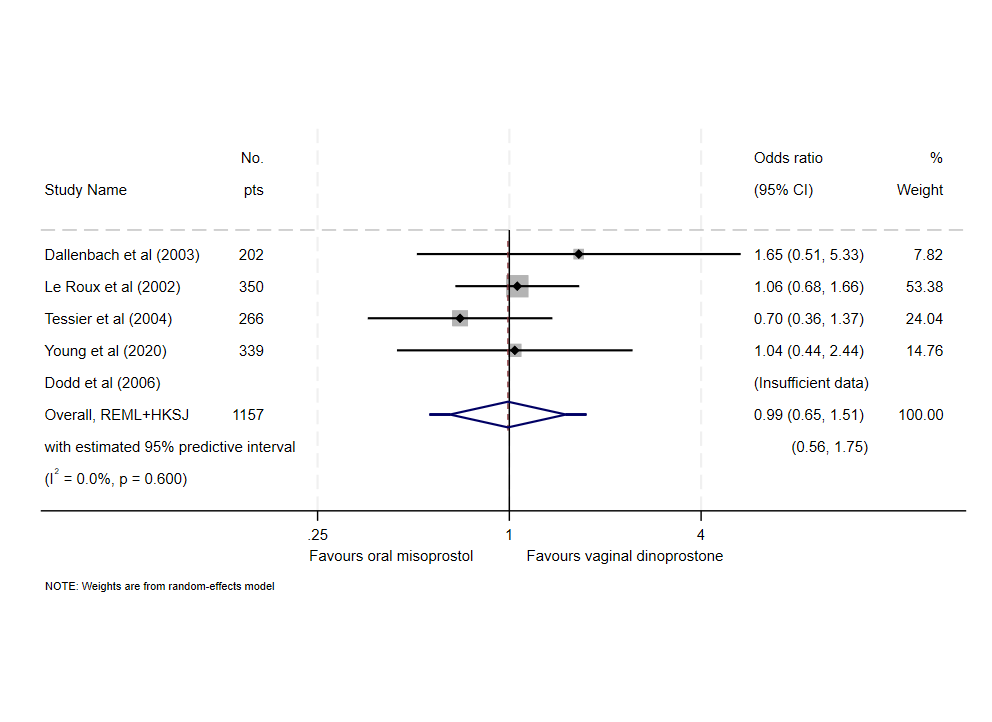
Figure

Figure S2G: Maternal infection


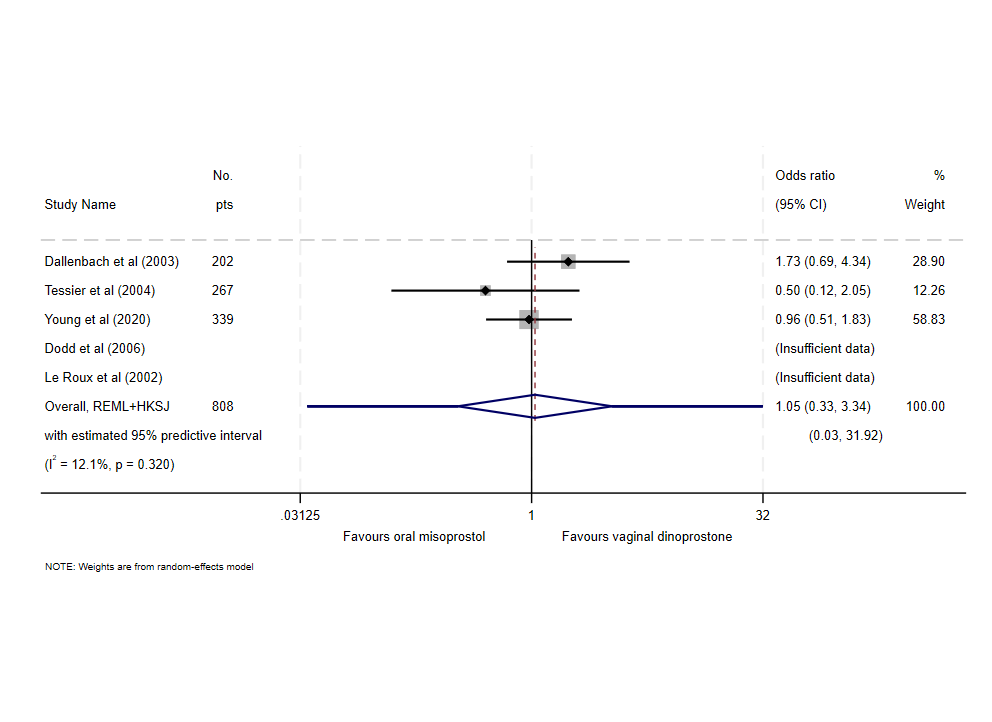


Figure S2H: Severe Postpartum Hemorrhage


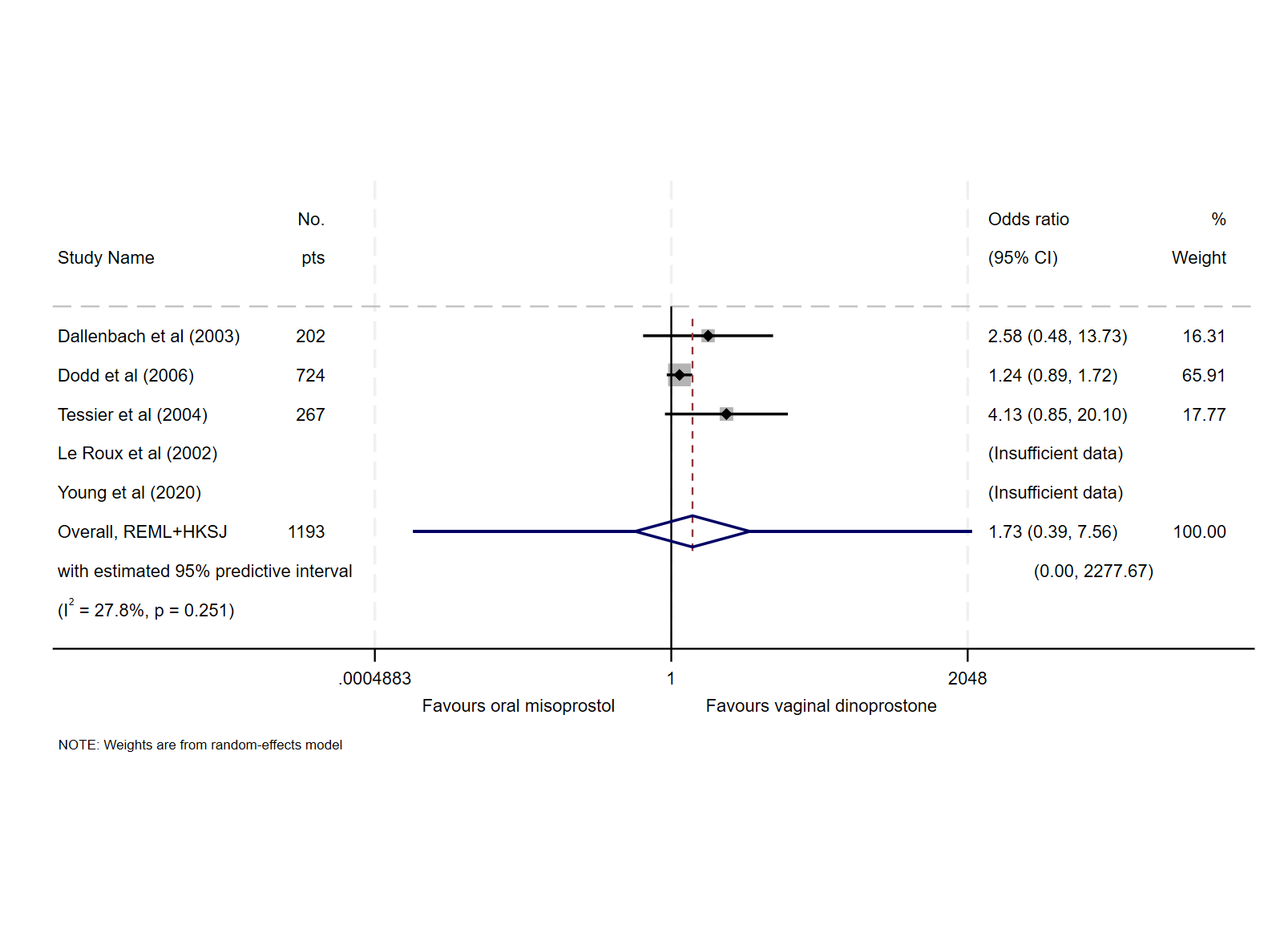


Figure S2I: Apgar score <7 at 5 minutes


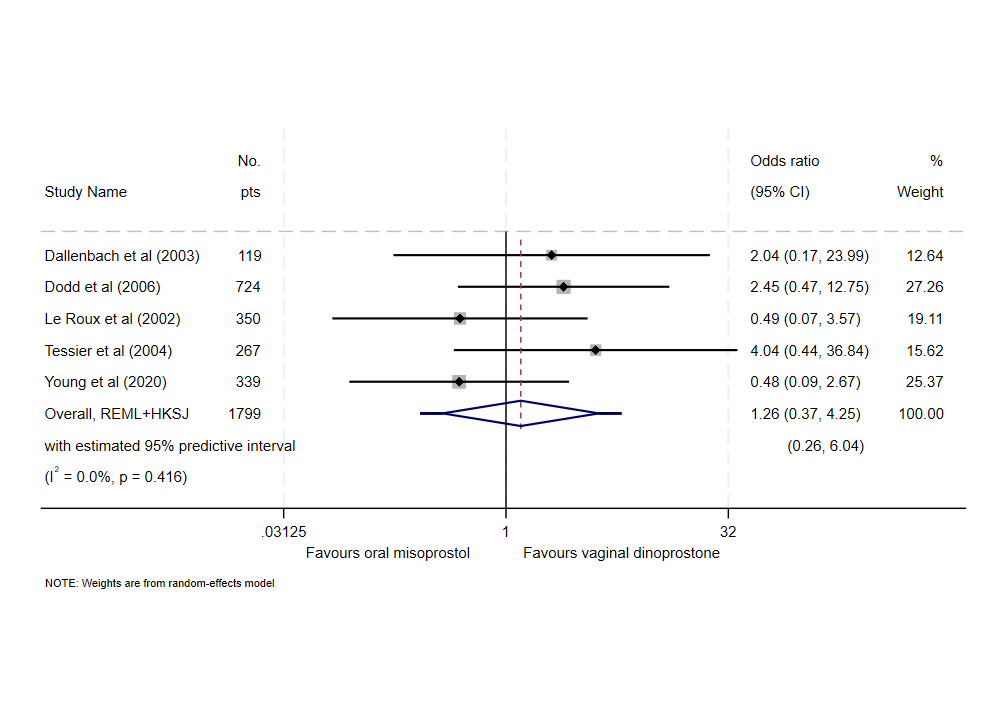


Figure S2J: NICU Admission


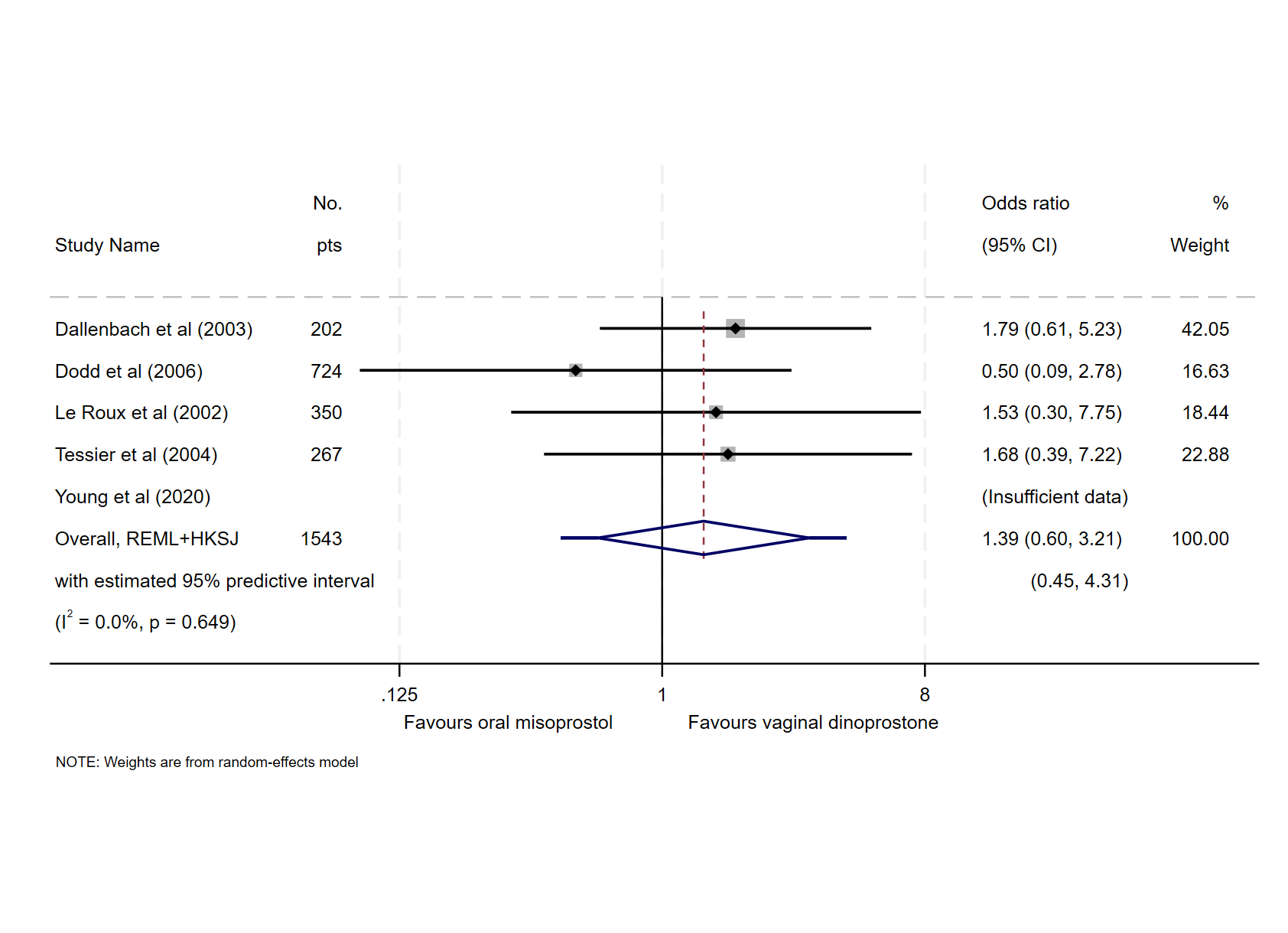


Figure S2K: Meconium-stained amniotic fluid


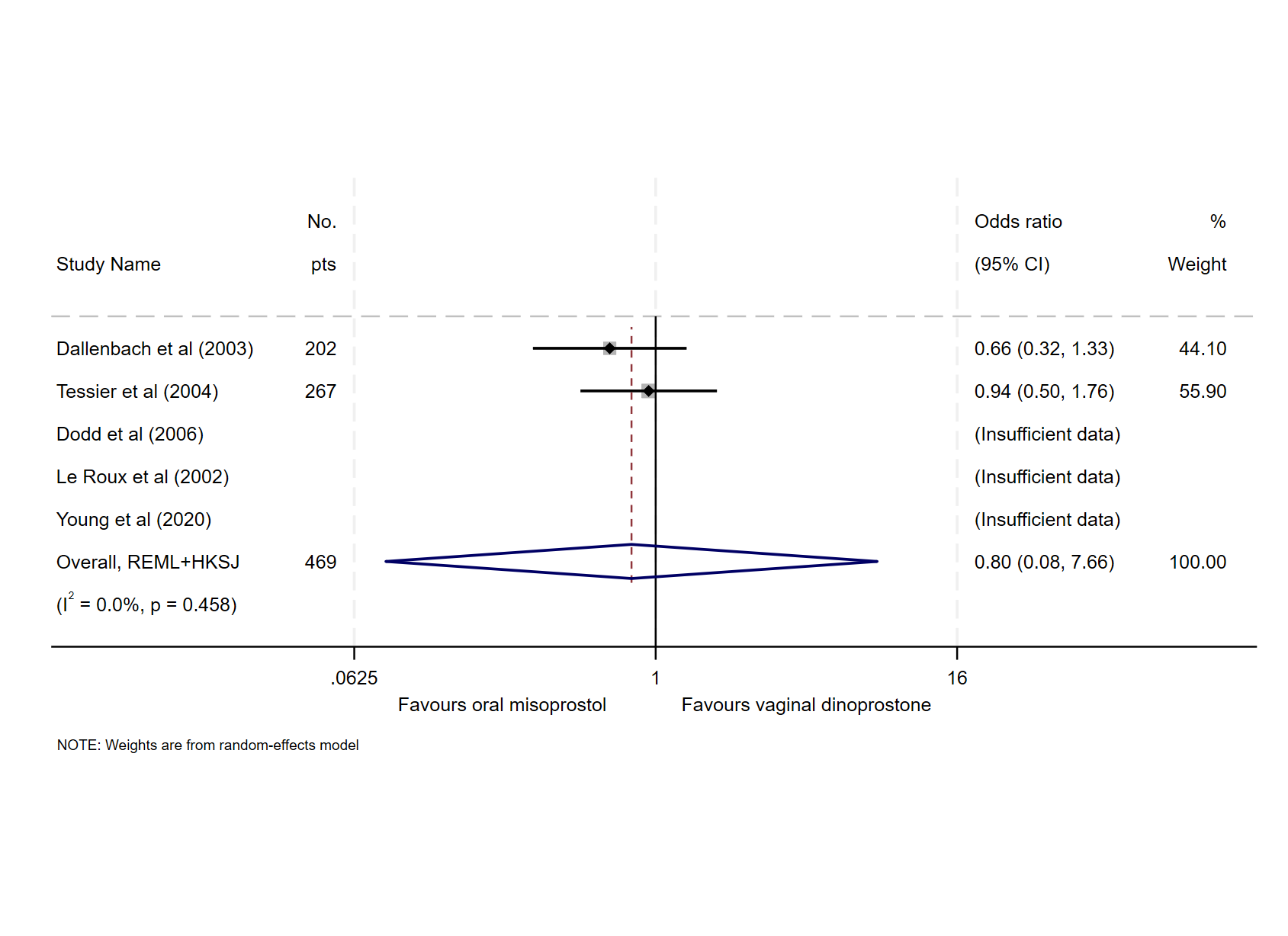


Figure S3: Stacked cumulative proportion plot of mode of delivery over time from labor induction to delivery. x-axis represents time (in h) on a logarithmic scale. y-axis represents cumulative percentage of deliveries.

*Note: The plot provides a descriptive representation of the cumulative distribution of deliveries over time. It does not account for competing risks or heterogeneity between studies.


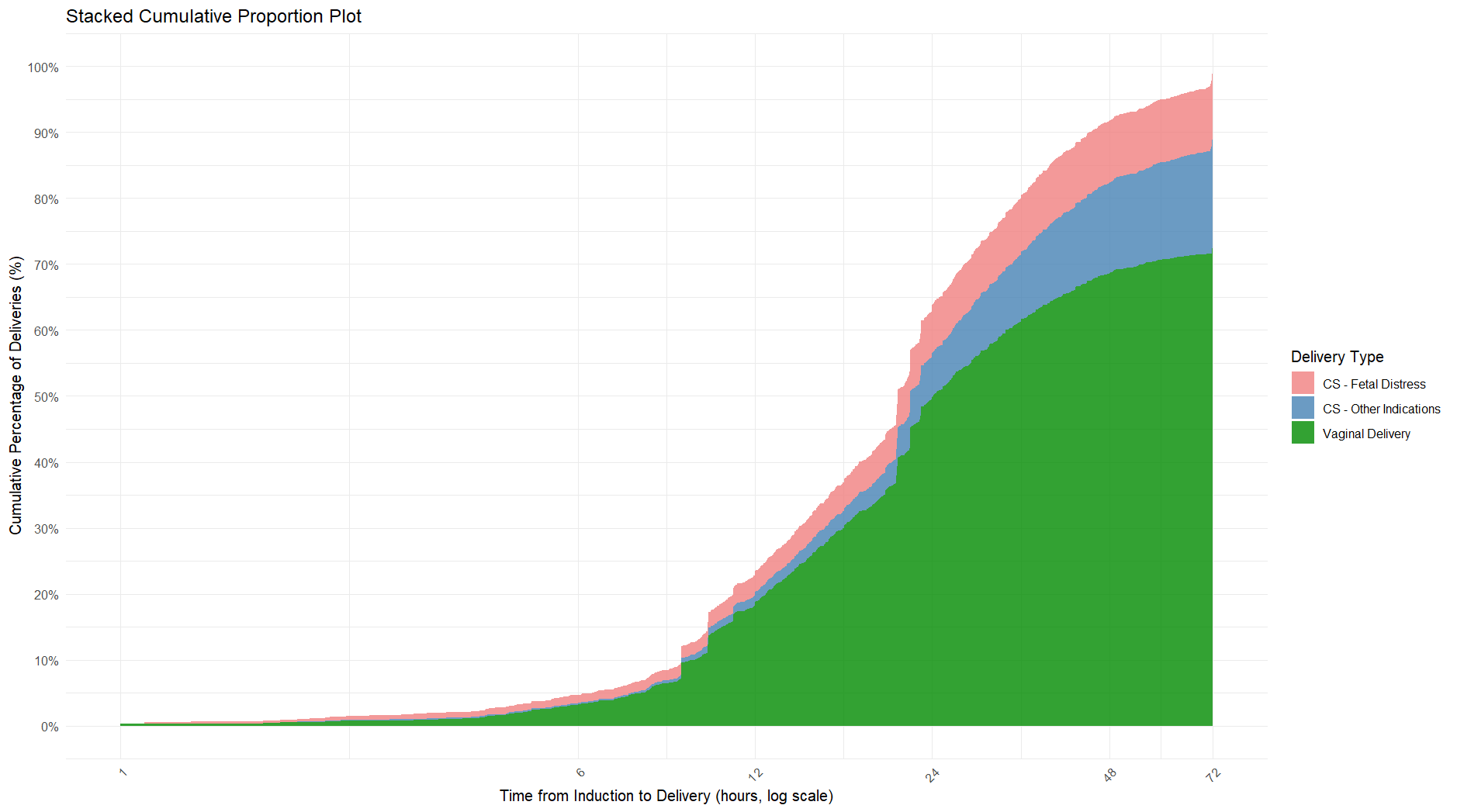


Figure S4: Forest plots comparing oral misoprostol vs vaginal dinoprostone for induction of labor according to rate of secondary outcomes, subdivided by individual participant data (IPD) and Trustworthiness in RAndomised Controlled Trials (TRACT) assessment. Threshold for trustworthiness was set as TRACT score ≤ 8, where + indicates no concern, ? indicates some concern/no information and – indicates major concern. Only first author is shown for each study. Not adjusted for maternal age or parity. Weights are from random-effects model.

Figure S4A: Post Partum Hemorrhage
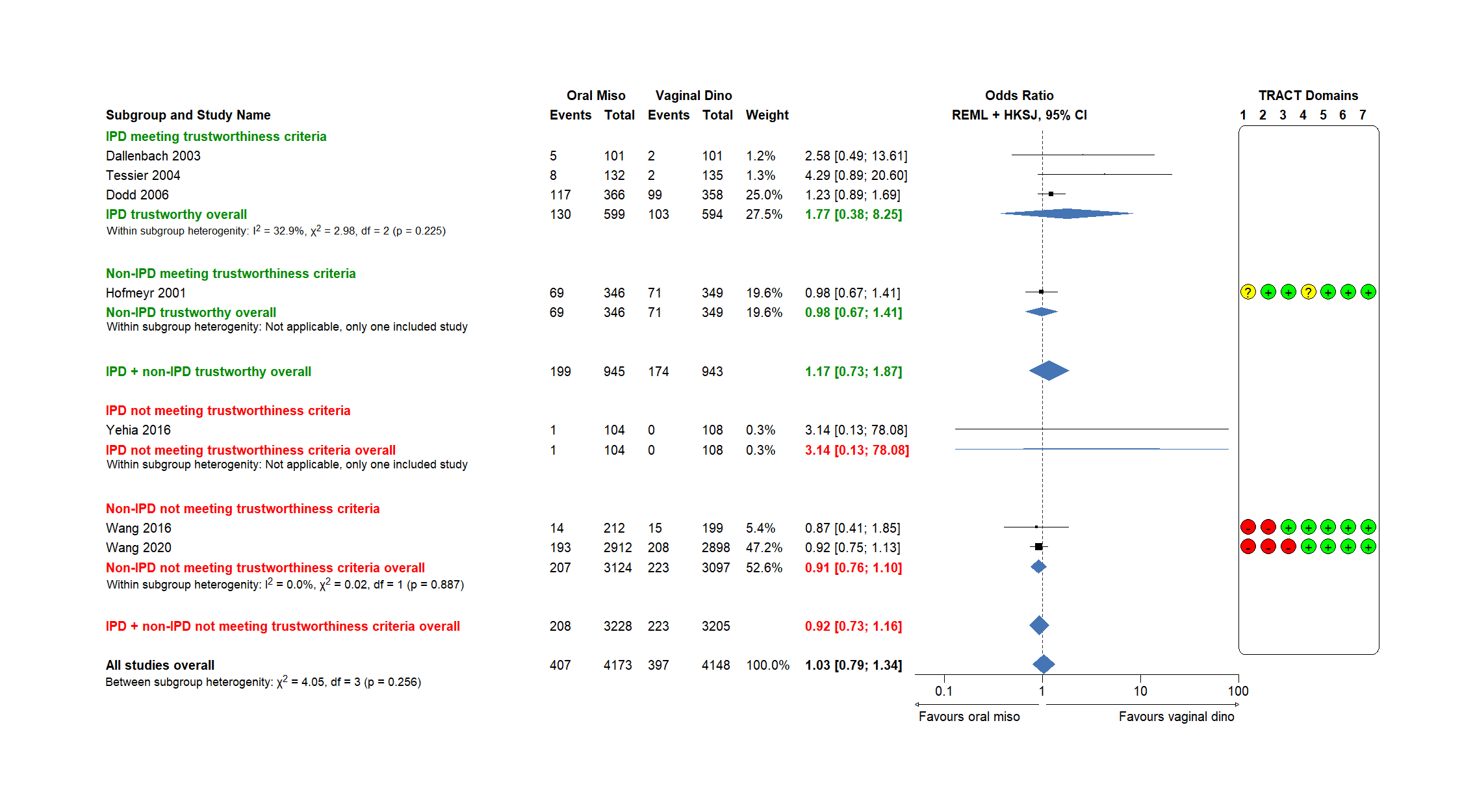


Figure S4B: APGAR <7 at 5 Minutes


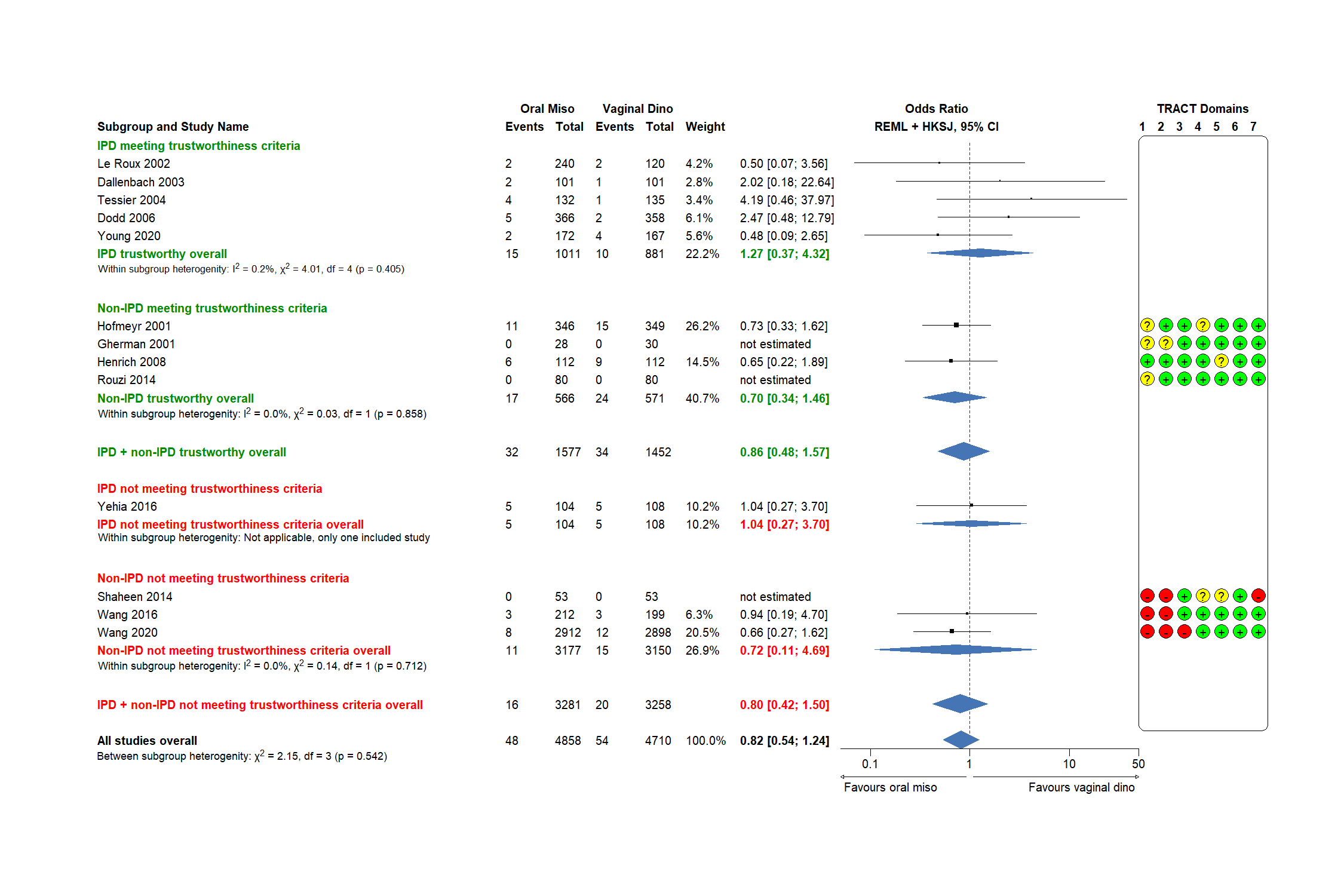


Figure S4C: NICU Admission


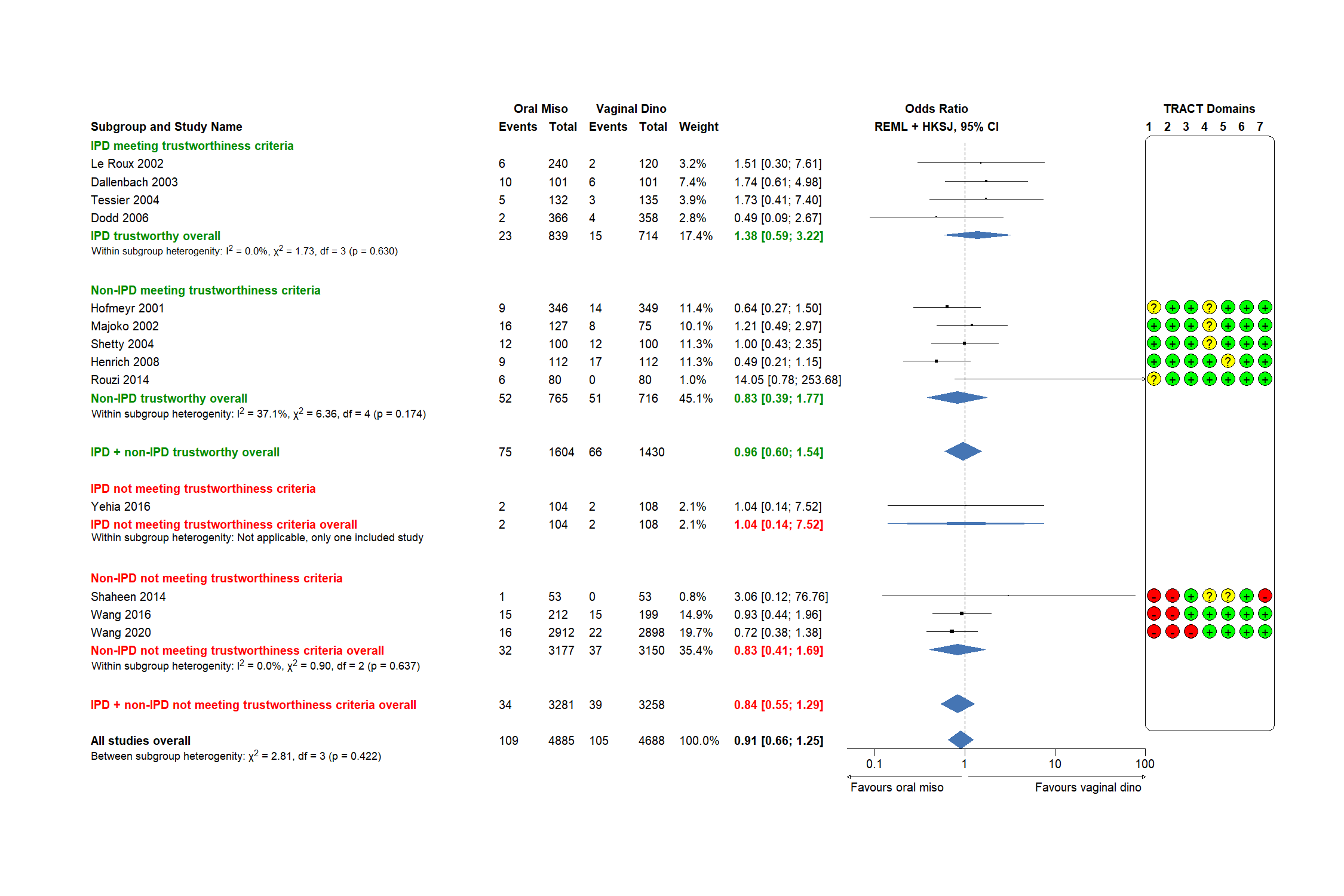


Figure S5: Funnel plot for primary outcome in all 18 randomized control trials for which individual participant data was requested.


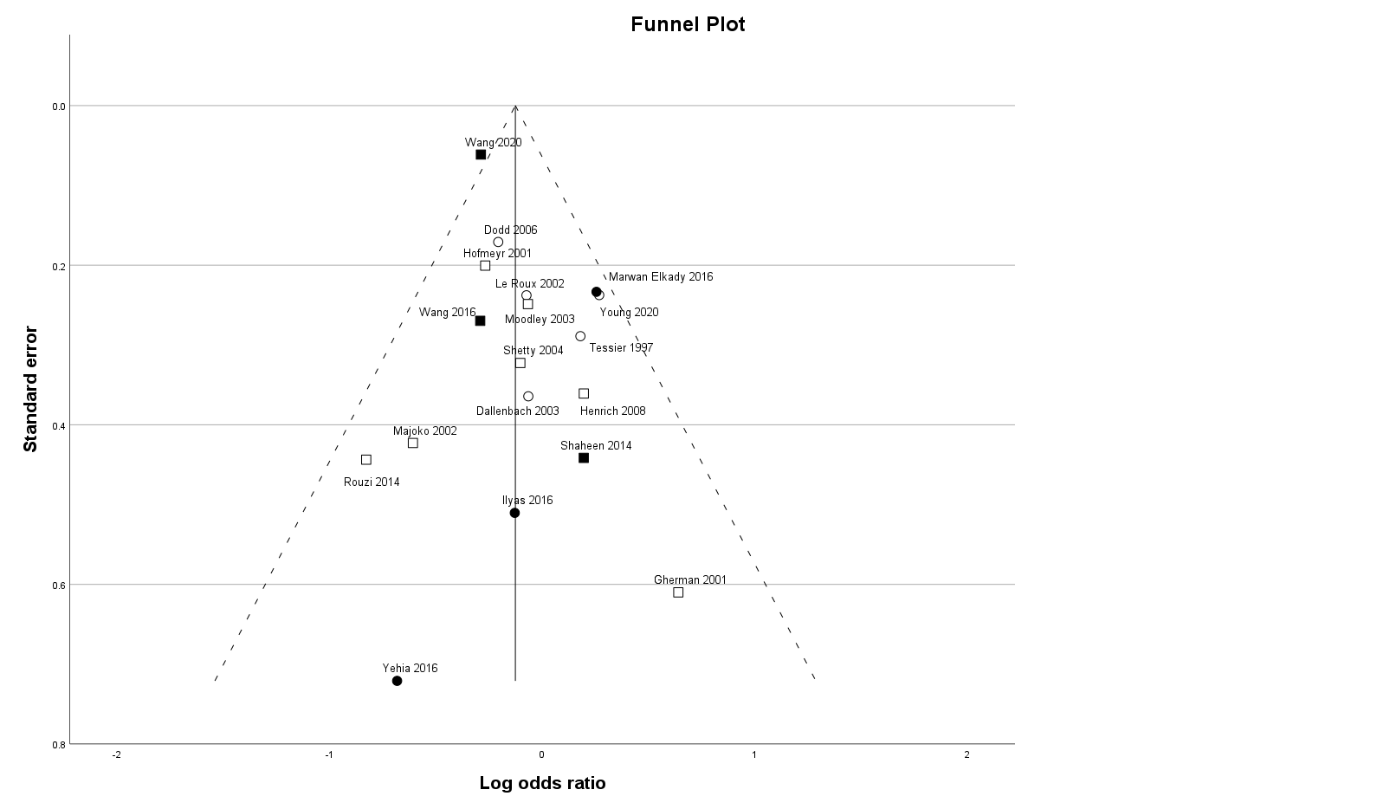


Trustworthy IPD

Not Trustworthy IPD

Trustworthy Non-IPD

Not Trustworthy Non-IPD

95% pseudo confidence intervals

Estimated overall effect size (observed studies)

## Supplementary Tables

Table S1: Search strategy

| **Source** | **Search** |
| --- | --- |
| CINAHL Plus | (MM “Misoprostol”) OR “misoprostol” OR PEG1 AND (MM “Dinoprostone”) OR “dinoprostone” OR PGE2 AND (MM “Cervix Dilataion and Effacement”) OR “cervical ripening” OR (MH “Labor, Induced+”) OR “(induce OR inducing OR induction) N3 (Labour OR labor)” |
| ClinicalTrials.gov | Labor, Induced \| Other terms: (induce OR inducing OR induction) OR (labour OR labor) OR cervical ripening \| (Misoprostol OR PGE1) OR (Dinoprostone OR PGE2) |
| Ovid Embase/Ovid Emcare/Ovid Medline | Misoprostol.mp. OR exp misoprostol/ OR PGE1.mp. OR exp prostaglanding E1/ AND dinoprostone.mp. OR exp prostagalandin E2/ OR PGE2.mp. AND ((induce or inducing or induction) adj3 (labour or labor)).mp. OR cervical ripending.mp or exp uterine cervix ripening/ OR labor induction.mp. or exp labor induction |
| Scopus | TITLE-ABS-KEY ((misoprostol OR pge1) AND (dinoprostone OR pge2) AND ((induce OR inducing OR induction) W/3 (labour OR Labor) OR “cervical ripening”)) |

Table S2: Trustworthiness in RAndomised Clinical Trials (TRACT) scoring system

| Rating per domain | Value assigned |
| --- | --- |
| No concerns | 0 |
| Some concerns/No information | 1 |
| Major concerns | 2 |

| Domain | | Total |
| --- | --- | --- |
| TRACT 1 | Absent or retrospective registration | _/6 |
|  | Sample size discrepancy of >15% |  |
|  | Absent or vague description of research ethics |  |
| TRACT 2 | < or 3 No. of authors | _/6 |
|  | Author retraction history |  |
|  | Large number of RCTs published |  |
| TRACT 3 | Implausible allocation concealment | _/4 |
|  | Illogical methodology |  |
| TRACT 4 | Fast recruitment | _/4 |
|  | Impossible timeframe |  |
| TRACT 5 | No loss to follow up | _/4 |
|  | Ideal numbers of losses to follow up/perfectly rounded |  |
| TRACT 6 | No or few baseline (<5) characteristics | _/8 |
|  | Implausible patient characteristics |  |
|  | Perfect balance/large differences between baseline characteristics |  |
|  | Prognostic factors are not reported |  |
| TRACT 7 | Larger effect size | _/6 |
|  | Conflicting information between outcomes |  |
|  | Change in primary outcome |  |
| Grand total | | **_/38** |

Table S3: Details of randomized controlled trials excluded from individual participant data (IPD) meta-analysis due to discrepancy between IPD received and published study

| *First author and year* | *Country* | *No. participants* | *Specific Variable/ Outcome with discrepancy* | *Details of discrepancy* | | | |
| --- | --- | --- | --- | --- | --- | --- | --- |
| *Elkady 2016^27^* | Egypt | 342 | All | Duplication of data – identical variable inputs of 103 participants to make a total of 342 participants | | | |
| *Ilyas 2016^28^* | Pakistan | 100 | Maternal age | No participants aged 29, 31, 33 and 34 in study | | | |
|  |  |  | Time to delivery |  | Misoprostol | Dinoprostone |  |
|  |  |  |  | Reported in Paper | 620.0 ± 115.7 | 930.0 ± 206.9 |  |
|  |  |  |  | Calculated from data | 660.0 ± 129.0 | 700.8 ± 162.5 |  |
| *Yehia 2016^93^* | Egypt | 212 | BMI | Random number generator “RAND()” found in formula for body mass index (BMI) | | | |

Table S4: Characteristics of randomized controlled trials for which enquiry for individual participant data (IPD) was sent but did not contribute to IPD meta-analysis

| First author and year | *Country* | *Recruitment period* | *Oral misoprostol* | *Vaginal dinoprostone* | | *Other group* | *No. participants* | *Inclusion criteria* | *Reason for not contributing to IPD* | *TRACT score (_/38)* |
| --- | --- | --- | --- | --- | --- | --- | --- | --- | --- | --- |
| Data not shared | | | | | | | | | | |
| Gherman 2001^17^ | America | Dec 7 1998 - Jun 15 1999 | 50µg tablets every 4hrs (max 6 doses) | | 0.5mg gel every 4hrs (max 6 doses) | - | 58 | Primip+multi, ≥24 weeks gestation, no PROM, BS ≤6, no previous CS | Data not available/data destroyed | 5 |
| Henrich 2008^18^ | Germany | Jan 2003 - Apr 2006 | 25µm, 50µg and then 100µg tablets 4hr intervals (max 175µg on the 1^st^ day and 300µg on the 2^nd^ day) | | 3mg tablet every 6hrs (max 9mg per day) | - | 224 | Primip+multi, ≥37 weeks gestation, no PROM, BS ≤6, no previous CS | Data not available/data destroyed | 6 |
| Hofmeyr 200123 | South Africa and United Kingdom | NS | Oral solution: 20µm increased to 40µg after 2 doses (UK) or after 3 doses (South Africa) every 2hrs (max NS) | | 2mg gel every 6hrs (max 4 doses) | - | 695 | Primip+multi, ≥34 weeks gestation, allows PROM, any BS, no previous CS | No response after initial response | 6 |
| Majoko 2002^19^ | Zimbabwe | 14mths, no dates provided | Oral solution: 10µg then doubled every 4hrs (max NS) | | 3mg pessary every 8hrs (max 2 doses) | Cervical dinoprostone (gel) + vaginal misoprostol (tablet) | 202 oral misoprostol and vaginal dinoprostol groups (total 406) | Primip+multi, >37 weeks gestation, PROM NS, any BS, no previous CS | Data not available/data destroyed | 5 |
| Moodley 2003^20^ | South Africa | NS | Oral solution: 20µg every 2hrs (max dose 80µm) | | 1mg gel every 6hrs (max 3 doses) | Vaginal + oral misoprostol | 296 oral misoprostol and vaginal dinoprostol groups (total 396) | Primip+multi (less than 6), gestation NS (“viable term or near-term”), allows PROM, BS <6, no previous CS | Data not available/data destroyed | 8 |
| Rouzi 2014^24^ | Saudi Arabia | Jan 2011 -  Jul 2012 | Oral solution: 20µg hourly for 2 doses, 30µg for 3 doses, 40µg then 50µg hourly, 60µg hourly for 4 doses | | 10mg pessary left for up to 24hrs | - | 160 | Primip+multi (less than 4), ≥34 weeks gestation, allows PROM, BS <6, no previous CS | No response after initial response | 6 |
| Shaheen 2014^22^ | Pakistan | Jan 1 2013 - Dec 31 2013 | 25µg tablet every 4hrs (max 4 doses) | | 2mg gel every 6hrs (max 2 doses) if Parity >3 1mg gel every 6hrs | - | 106 | Primip+multi (“Grandmultiparas”, not defined, excluded), 37-41 weeks gestation, allows PROM, BS <6, no previous CS | No response | 12 |
| Shetty 2004^21^ | UK | Oct 2001 - Feb 2002 | 100µg tablet every 4hrs (max 5 doses) | | 3mg tablet every 6hrs (max 2 doses) | - | 200 | Primip+multi (parity ≥5 excluded), ≥37 weeks gestation, no PROM, BS <8, no previous CS | Data not available/data destroyed | 5 |
| Wang 2016^25^ | China | Jan 2014 - Oct 2014 | Oral solution: 20µg hourly for 2 doses,30µg hourly for 3 doses, then 1 dose 40µg 1.5hrs, 1 dose 50µg 2hrs, 60µg every 2hrs for 2 doses | | NS | - | 411 | Primip, ≥36 weeks gestation, allows PROM, BS <6, no previous uterine scar | No response after initial response | 10 |
| Wang 2020^26^ | China | Jun 1 2016 - Oct 1 2019 | Oral solution: 20µg hourly for 2 doses,30µg hourly for 3 doses, then 1 dose 40µg 1.5hrs, 1 dose 50µg, whole administration cycle repeated after 6hr interval | | 10mg pessary (max time NS) | - | 5,810 | Primip, ≥36 weeks gestation, allows PROM, BS <7, no previous uterine scar | No response after initial response | 10 |
| Data shared but excluded after data enquiry | | | | | | | | | | |
| Elkady 2016^27^ | Egypt | NS | 25µg every 2hrs (max 4 doses) | | 3mg tablet every 6hrs (max 2 doses) | - | 342 | NS | - | - |
| Ilyas 2016^28^ | Pakistan | Jan 2011 - Jun 2011 | 50µg every 4hrs (max 3 doses) | | 0.5mg gel every 6hrs (max 2 doses) | - | 100 | Primip+multi, 37-41 weeks gestation, PROM required, BS NS, no previous CS | - | - |
| Yehia 2016^29^ | Egypt | Jun 2013 - Aug 2014 | Oral solution: 20µg every 2hrs for 2 doses, then 1 dose of: 40µg, 45µg, 50µg, 60µg every 2hrs | | 3mg tablet every 6hrs (max 2 doses) | - | 212 | Primip+multi, >37 weeks gestation, no PROM, BS <8, no previous CS | - | - |

NS= not stated. PROM= premature rupture of membranes. BS= bishop score. CS= caesarean section. Primip= primiparous women. Multip = multiparous women

Table S5: Baseline characteristics of participants in randomized controlled trials included in individual participant data meta-analysis, stratified by individual study

| Characteristics | Oral Misoprostol | Vaginal Dinoprostone |
| --- | --- | --- |
| Dallenbach 2003^34^ | | |
|  | N = 101 | N = 101 |
| Maternal age (years) mean (SD) | 30.5 (5.0) | 29.9 (5.5) |
| BMI kg/m^2^ * mean (SD) | - | - |
| Gestational age (weeks) mean (SD) | 39.8 (1.3) | 39.8 (1.4) |
| Initial modified bishops score mean (SD) | 2.87 (1.1) | 3.25 (1.0) |
| Parity n/N (%) |  |  |
| 0 | 58/101 (57.4%) | 61/101 (60.4%) |
| ≥1 | 43/101 (42.6%) | 40/101 (39.6%) |
| Indication for IOL n/N (%) |  |  |
| Other/unknown | 21/101 (20.8%) | 16/101 (15.8%) |
| Hypertensive disorders | 1/101 (1.0%) | 2/101 (2.0%) |
| Post-term pregnancy | 41/101 (41.6%) | 49/101 (48.5%) |
| Diabetes/gestational diabetes | 1/101 (1.0%) | 1/101 (1.0%) |
| Oligohydramnios | 2/101 (2.0%) | 2/101 (2.0%) |
| Fetal growth restriction | 3/101 (3.0%) | 1/101 (1.0%) |
| Pre-labour rupture of membranes | 18/101 (17.8%) | 25/101 (24.8%) |
| Decreased fetal movements | 0/101 (0.0%) | 0/101 (0.0%) |
| Elective/maternal request | 1/101 (1.0%) | 1/101 (1.0%) |
| Antepartum haemorrhage | 0/101 (0.0%) | 0/101 (0.0%) |
| >1 indication | 12/101 (11.9%) | 4/101 (4.0%) |
| *No data for BMI |  |  |
| Dodd 2006^30^ | | |
|  | N = 358 | N = 366 |
| Maternal age (years) mean (SD) | 27.9 (5.6) | 27.9 (5.5) |
| BMI kg/m^2^ * mean (SD) | 28.0 (6.6) | 28.0 (6.2) |
| Gestational age (weeks) mean (SD) | 40.0 (1.2) | 40.0 (1.3) |
| Initial modified bishops score mean (SD) | 2.94 (1.3) | 3.12 (1.3) |
| Parity n/N (%) |  |  |
| 0 | 210/358 (58.7%) | 217/366 (59.3%) |
| ≥1 | 148/358 (41.3%) | 149/366 (40.7%) |
| Indication for IOL n/N (%) |  |  |
| Other/unknown | 69/358 (19.3%) | 56/366 (15.3%) |
| Hypertensive disorders | 65/358 (18.2%) | 66/366 (18.0%) |
| Post-term pregnancy | 160/358 (44.7%) | 156/366 (42.6%) |
| Diabetes/gestational diabetes | 21/358 (5.9%) | 35/366 (9.6%) |
| Oligohydramnios | 1/358 (0.3%) | 1/366 (0.3%) |
| Fetal growth restriction | 19/358 (5.3%) | 13/366 (3.6%) |
| Pre-labour rupture of membranes | 0/358 (0.0%) | 0/366 (0.0%) |
| Decreased fetal movements | 0/366 (0.0%) | 0/366 (0.0%) |
| Elective/maternal request | 0/358 (0.0%) | 0/366 (0.0%) |
| Antepartum haemorrhage | 0/358 (0.0%) | 0/366 (0.0%) |
| >1 indication | 23/358 (6.4%) | 39/366 (10.7%) |
| *158 missing for BMI |  |  |
| Le Roux 2002^33^ | | |
|  | N = 120 | N =240 |
| Maternal age (years)* mean (SD) | 28.1 (6.1) | 27.6 (6.0) |
| BMI kg/m^2^ * mean (SD) | - | - |
| Gestational age (weeks) mean (SD) | 38.6 (2.3) | 39.0 (2.3) |
| Initial modified bishops score* mean (SD) | 3.45 (1.5) | 3.51 (1.7) |
| Parity* n/N (%) |  |  |
| 0 | 44/119 (36.7%) | 101/240 (42.1%) |
| ≥1 | 75/119 (62.5%) | 139/240 (57.9%) |
| Indication for IOL n/N (%) |  |  |
| Other/unknown | 15/120 (12.5%) | 32/240 (13.3%) |
| Hypertensive disorders | 53/120 (44.2%) | 97/240 (40.4%) |
| Post-term pregnancy | 25/120 (20.8%) | 62/240 (25.8%) |
| Diabetes/gestational diabetes | 12/120 (10.0%) | 15/240 (6.3%) |
| Oligohydramnios | 6/120 (5.0%) | 15/240 (6.3%) |
| Fetal growth restriction | 4/120 (3.3%) | 8/240 (3.3%) |
| Pre-labour rupture of membranes | 0/120 (0.0%) | 0/240 (0.0%) |
| Decreased fetal movements | 0/120 (0.0%) | 0/240 (0.0%) |
| Elective/maternal request | 0/120 (0.0%) | 0/240 (0.0%) |
| Antepartum haemorrhage | 3/120 (2.5%) | 3/240 (1.3%) |
| >1 indication | 2/120 (1.7%) | 8/240 (3.3%) |
| *9 missing for maternal age, no data for BMI, 4 missing for initial modified bishop score, 1 missing for parity |  |  |
| Tessier 1997^31^ | | |
|  | N = 135 | N = 132 |
| Maternal age (years) mean (SD) | 30.9 (5.1) | 30.3 (5.4) |
| BMI kg/m^2^ * mean (SD) | - | - |
| Gestational age (weeks) mean (SD) | 39.5 (1.8) | 39.3 (2.0) |
| Initial modified bishops score* mean (SD) | 5.3 (2.0) | 5.0 (2.0) |
| Parity n/N (%) |  |  |
| 0 | 95/135 (70.4%) | 93/132 (70.5%) |
| ≥1 | 40/135 (29.6%) | 39/132 (29.5%) |
| Indication for IOL n/N (%) |  |  |
| Other/unknown | 18/135 (13.3%) | 18/132 (13.6%) |
| Hypertensive disorders | 11/135 (8.1%) | 9/132 (6.8%) |
| Post-term pregnancy | 55/135 (40.7%) | 49/132 (37.1%) |
| Diabetes/gestational diabetes | 3/135 (2.2%) | 3/132 (2.3%) |
| Oligohydramnios | 4/135 (3.0%) | 4/132 (3.0%) |
| Fetal growth restriction | 2/135 (1.5%) | 8/132 (6.1%) |
| Pre-labour rupture of membranes | 10/135 (7.4%) | 14/132 (10.6%) |
| Decreased fetal movements | 0/135 (0.0%) | 1/132 (0.8%) |
| Elective/maternal request | 0/135 (0.0%) | 1/132 (0.8%) |
| Antepartum haemorrhage | 0/135 (0.0%) | 0/132 (0.0%) |
| >1 indication | 32/135 (23.7%) | 25/132 (18.9%) |
| *No data for BMI, 1 missing for initial modified bishop score |  |  |
| Young 2020^32^ | | |
|  | N = 167 | N = 172 |
| Maternal age (years) mean (SD) | 28.8 (5.2) | 28.8 (5.0) |
| BMI kg/m^2^ * mean (SD) | 34.1 (6.7) | 33.8 (5.4) |
| Gestational age (weeks) mean (SD) | 40.0 (1.5) | 40.0 (1.5) |
| Initial modified bishops score* mean (SD) | 3.8 (1.9) | 4.2 (2.1) |
| Parity n/N (%) |  |  |
| 0 | 108/167 (64.7%) | 107/172 (62.2%) |
| ≥1 | 59/167 (35.3%) | 65/172 (37.8%) |
| Indication for IOL n/N (%) |  |  |
| Other/unknown | 5/167 (3.0%) | 7/172 (4.1%) |
| Hypertensive disorders | 28/167 (16.8%) | 30/172 (17.4%) |
| Post-term pregnancy | 68/167 (40.7%) | 71/172 (41.3%) |
| Diabetes/gestational diabetes | 0/167 (0.0%) | 2/172 (1.2%) |
| Oligohydramnios | 8/167 (4.8%) | 8/172 (4.7%) |
| Fetal growth restriction | 0/167 (0.0%) | 4/172 (2.3%) |
| Pre-labour rupture of membranes | 10/167 (6.0%) | 13/172 (7.6%) |
| Decreased fetal movements | 2/167 (1.2%) | 1/172 (0.6%) |
| Elective/maternal request | 5/167 (3.0%) | 3/172 (1.7%) |
| Antepartum haemorrhage | 2/167 (1.2%) | 0/172 (0.0%) |
| >1 indication | 39/167 (23.4%) | 33/172 (19.2%) |
| *149 missing for BMI, 12 missing for initial modified bishop score |  |  |

Table S6: Subgroup analysis for vaginal delivery (vaginal dinoprostone vs oral misoprostol) and composite adverse maternal outcome and composite adverse perinatal outcome (oral misoprostol vs vaginal dinoprostone)

| Subgroup | *No. of trials* | *No. of women* | *iOR (95% CI)* | *I^2^ (p-value)* | *Analysis method* |
| --- | --- | --- | --- | --- | --- |
| Vaginal Delivery | | | | | |
| Parity | 5 | 1,891 | 0.92 (0.32-2.23) | 36.9% | Two-stage |
| Gestational age | 5 | 1,891 | 0.91 (0.86-0.97) | 0.0% | Two-stage |
| Pre-term (<37 weeks)  Term (37-40 weeks)  Post-term (>40 weeks) | -  -  - | 48  978  714 | 1.18 (0.41-3.42)  0.89 (0.68-1.16)  0.73 (0.36-1.48) | -  -  - | -  -  - |
| PROM | 1 | 202 | 0.82 (0.13-4.98) | 0.0% | Two-stage |
| Age | 5 | 1,891 | 1.01 (0.96-1.07) | 5.0% | Two-stage |
| BMI | 2 | 1,045 | 1.00 (0.78-1.28) | 0.0% | Two-stage |
| Baseline bishop score | 5 | 1,874 | 1.02 (0.94-1.12) | 0.0% | Two-stage |
| Maternal Adverse Events | | | | | |
| Parity | 2 | 991 | 1.65 (0.07-41.79) | 0.0% | Two-stage |
| Gestational age | 3 | 1,193 | 1.08 (0.99-1.17) | 0.0% | Two-stage |
| PROM | 2 | 469 | 0.27 (0.06-1.26) | 0.0% | Two-stage |
| Age | 3 | 1,193 | 0.98 (0.85-1.12) | 0.0% | Two-stage |
| BMI | 2 | 991 | 0.98 (0.84-1.14) | 0.0% | Two-stage |
| Baseline bishop score | 3 | 1,192 | 0.90 (0.63-1.29) | 0.0% | Two-stage |
| Perinatal Adverse Events | | | | | |
| Parity | 4 | 1,168 | 1.27 (0.11-14.20) | 38.1% | Two-stage |
| Gestational age | 5 | 1,892 | 1.16 (0.70-1.90) | 22.6% | Two-stage |
| PROM | 2 | 469 | 0.33 (0.00-22.36) | 0.0% | Two-stage |
| Age | 5 | 1,892 | 1.09 (0.90-1.32) | 41.3% | Two-stage |
| BMI | 2 | 991 | 1.13 (0.74-1.73) | 0.0% | Two-stage |
| Baseline bishop score | 5 | 1,875 | 0.97 (0.47-1.98) | 47.3% | Two-stage |

iOR: interact odds ratio; PROM: premature rupture of membranes

| **Study** | **Governance** | | | **Author Group** | | | **Plausibility of Intervention Usage** | | **Timeframe** | | **Drop-Out Rates** | | **Baseline Characteristics** | | | | **Outcomes** | | | **Overall Score (_/38)** | **Trustworthy** |
| --- | --- | --- | --- | --- | --- | --- | --- | --- | --- | --- | --- | --- | --- | --- | --- | --- | --- | --- | --- | --- | --- |
| *Gherman 2001^17^* | SC/NI | SC/NI | SC/NI | NC | SC/NI | NC | NC | NC | NC | NC | NC | NC | NC | NC | NC | NC | NC | NC | SC/NI | 5 | Yes |
|  | Larger than sample size required | | | Possible retracted paper for 1 author | | |  | |  | |  | |  | | | |  | | |  |  |
| *Henrich 2008^18^* | SC/NI | SC/NI | SC/NI | NC | NC | NC | NC | NC | NC | NC | SC/NI | SC/NI | NC | NC | NC | NC | NC | NC | SC/NI | 5 | Yes |
|  | No sample size calculation | | |  | | |  | |  | | No mention of loss to follow up, the same number of participants in each group | |  | | | |  | | |  |  |
| *Hofmeyr 2001^23^* | SC/NI | SC/NI | SC/NI | NC | NC | NC | NC | NC | SC/NI | SC/NI | NC | NC | NC | NC | NC | NC | NC | NC | SC/NI | 6 | Yes |
|  | Larger than sample size required | | |  | | |  | | No information on timeframe | |  | |  | | | |  | | |  |  |
| *Majoko 2002^19^* | SC/NI | SC/NI | SC/NI | NC | NC | NC | NC | NC | NC | SC/NI | NC | NC | NC | NC | NC | NC | NC | NC | SC/NI | 5 | Yes |
|  |  | | |  | | |  | | No information on timeframe | |  | |  | | | |  | | |  |  |
| *Moodley 2003^20^* | SC/NI | SC/NI | SC/NI | MC | NC | NC | NC | NC | SC/NI | SC/NI | NC | NC | NC | NC | NC | NC | NC | NC | SC/NI | 8 | Yes |
|  | The same sample size that was calculated | | | Only 3 authors | | |  | | No information on timeframe | |  | |  | | | |  | | |  |  |
| *Rouzi 2014^24^* | MC | SC/NI | SC/NI | NC | NC | NC | NC | NC | NC | NC | NC | NC | NC | NC | NC | NC | SC/NI | NC | SC/NI | 6 | Yes |
|  | No registration after 2010, Sample size smaller than calculated | | |  | | |  | |  | |  | |  | | | |  | | |  |  |
| *Shaheen 2014^22^* | MC | SC/NI | SC/NI | MC | NC | NC | NC | NC | NC | SC/NI | SC/NI | SC/NI | NC | NC | NC | NC | NC | MC | SC/NI | 12 | No |
|  | No registration after 2010 | | | Only 2 authors | | |  | | Only 6mths between end of study to publication | | The same number of participants in each group | |  | | | | Number of vaginal delivery and caesarean section does not add up to 100% | | |  |  |
| *Shetty 2004^21^* | SC/NI | SC/NI | SC/NI | NC | NC | NC | NC | NC | SC/NI | NC | NC | NC | NC | NC | NC | NC | NC | NC | SC/NI | 5 | Yes |
|  |  | | |  | | |  | | 200 participants recruited in 5 months | |  | |  | | | |  | | |  |  |
| *Wang 2016^25^* | MC | SC/NI | MC | NC | MC | NC | NC | NC | NC | NC | NC | NC | NC | NC | NC | NC | NC | NC | SC/NI | 10 | No |
|  | No registration after 2010, no study size calculation, no ethics mentioned | | | 2 authors have 1 paper retracted each | | |  | |  | |  | |  | | | | Statistical mistakes | | |  |  |
| *Wang 2020^26^* | MC | SC/NI | SC/NI | MC | NC | NC | MC | NC | NC | NC | NC | NC | NC | NC | NC | NC | SC/NI | NC | SC/NI | 10 | No |
|  | No registration after 2010, hospital ethics only | | | Only 6 authors | | | No concealment | | Unfeasible large sample size | |  | |  | | | | Statistical mistakes | | |  |  |

Table S7: Trustworthiness in RAndomised Clinical Trials (TRACT) scores for randomized control studies that did not share individual participant data

NC: no concerns; SC/NI: some concerns/no information; MC: major concerns
